# Fate identification and management strategies of non-recyclable plastic waste through the integration of material flow analysis and leakage hotspot modeling

**DOI:** 10.1038/s41598-022-20594-w

**Published:** 2022-09-29

**Authors:** Aprilia Nidia Rinasti, Indradhi Faisal Ibrahim, Kavinda Gunasekara, Thammarat Koottatep, Ekbordin Winijkul

**Affiliations:** 1 Marine Plastics Abatement Program, School of Environment, Resources, and Development, Asian Institute of Technology, 58 Moo 9, Paholyothin Road, Pathum Thani, 12120 Thailand; 2 Geoinformatics Center, Asian Institute of Technology, 58 Moo 9, Paholyothin Road, Pathum Thani, 12120 Thailand; 3 Environmental Engineering and Management Program, School of Environment, Resources, and Development, Asian Institute of Technology, 58 Moo 9, Paholyothin Road, Pathum Thani, 12120 Thailand

**Keywords:** Environmental sciences, Hydrology

## Abstract

Low priority on waste management has impacted the complex environmental issue of plastic waste pollution, as evident by results of this study where it was found that 24.3% of waste generation in Jakarta and Bandung is emitted into the waterway due to the high intensity of human activity in the urban area. In this study, we investigated the viable integration between material flow analysis and leakage hotspot modeling to improve management strategies for plastic pollution in water systems and open environments. Using a multi-criteria assessment of plastic leakage from current waste management, a material flow analysis was developed on a city-wide scale defining the fate of plastic waste. Geospatial analysis was assigned to develop a calculation for identification and hydrological analysis while identifying the potential amount of plastic leakage to the river system. The results show that 2603 tons of plastic accumulated along the mainstream of the Ciliwung River on an annual basis, and a high-density population like that in Bandung discarded 1547 tons in a one-year period to the Cikapundung River. The methods and results of this study are applicable towards improving the control mechanisms of river rejuvenation from plastic leakage by addressing proper management in concentrated locations.

## Introduction

Plastic waste, which is estimated to double by 2050, has grown rapidly across nations and overwhelmed waste management systems^1^. One option to reduce existing and upcoming plastic waste is by recycling, which also reduces the usage of oil, carbon dioxide emissions, and disposal^2^. Although recycling is an available method to mitigate the impacts of the plastic industry^3^, only approximately 9% of plastic waste has been recycled^1^. The recycling rate also decreases by the presence of non-recyclable plastic i.e., thermosetting plastic^4^, thermoset composite^5^, and multilayered plastic^6^. Moreover, some recycled plastics are not as environmentally friendly as virgin plastics due to additives in the products such as stabilizers and flame retardants^6^. In addition, although many plastics are classified as recyclable, the cost of recycling is not always economically valuable since it’s driven by both supply and demand in the recycling market^7^.

Apart from issues with recycling, excessive amounts of plastic waste in the environment have been implicated by its presence in the water system on the sub-basin level^8,9^ and as riverine plastics^10^. To address and quantify this issue, approaches have been developed by utilizing spatial analysis using Geographic Information Systems (GIS). GIS approaches are developing through three pillars of sustainability identification aspects^11,12^: developing waste management scenarios by monetization of the economic condition^13^^,^^14^, implying seasonal changes^15^, and adapting the hydrological model^16^. Approaches to identify the amount of plastic waste leakage have also been developed with remote sensing monitoring technologies in aquatic and terrestrial environments using Unmanned Aerial Vehicle^17,18^ and satellite-based monitoring^19–22^. Further, some approaches have been designed towards integrating waste management systems with spatial analysis using GIS^11,23,24^. Based on these, the discarded plastic waste in open environments has been identified, although the existing conditions do not allow the source identification.

Indonesia has issued Presidential Decree No.97/2017 (National Waste Management Policy and Strategy)^25^, aiming at 30% waste reduction and 70% waste handling by 2025. Further, Presidential Decree No.83/2018 (Marine Debris Management)^26^ has also been enforced to reduce 70% of marine plastic debris by 2025. To achieve the targets, the country needs to double the current recycling capacity (more 975,000 tons of recycled plastic per year) by 2025^27^. However, recyclers often cannot obtain appropriate plastics for their industry^28^. For solutions, Indonesia is sanguine to implementing design for sustainability (DfS)^29^ and developing plastic waste road construction mixtures^30^. Globally, chemical recycling and thermal recycling methods, such as gasification and refuse derived fuel (RDF)^31^ and plasma pyrolysis technology^6^ are example methods to manage plastic that cannot be recycled into raw materials.

Although efforts on recycling plastic waste have been implemented, some issues have not been addressed where the leakage term also accommodates recycling stagnancy^7^. Therefore, a calculation through each stage of the waste management system spatially would be beneficial to quantify the presence of plastic waste in the environment. One of the closest approaches made has been to improve the consumption level country^14^ and city-wide data on waste management^12^. To comprehend material shifting analysis on the existing waste management system, we integrated the concept of material flow analysis and spatial domain study to enhance the concentrated leakage amount as one of the systems approaches to integrate solid waste management. Waste management stages imply an increment study on the assessment of waste^32^, which is also suitable to integrate with spatial analysis using GIS^33,34^.

In this study, we defined initial non-recyclable plastic waste (NRPW) as plastic waste that cannot be recycled by current recycling practices in the specified area because of technical barriers such as the absence of technology, the presence of complex plastic polymer structures, and/or environmental pollution caused by recycling activity. Meanwhile, poor management practices, such as no-segregation waste systems, open burning, and littering, can also turn plastic into a non-recyclable due to low quality or low economic value. We calculated the amount of plastic waste leakage by using a material flow analysis assessment and identified NRPW according to the definition and improved the information on the specific leakage locations. The main objective of this study was to improve the measurement of the NRPW leakage pathway from the source to the destination.

## Results

### Material flow analysis of plastic waste (2021)

**Figure 1:**
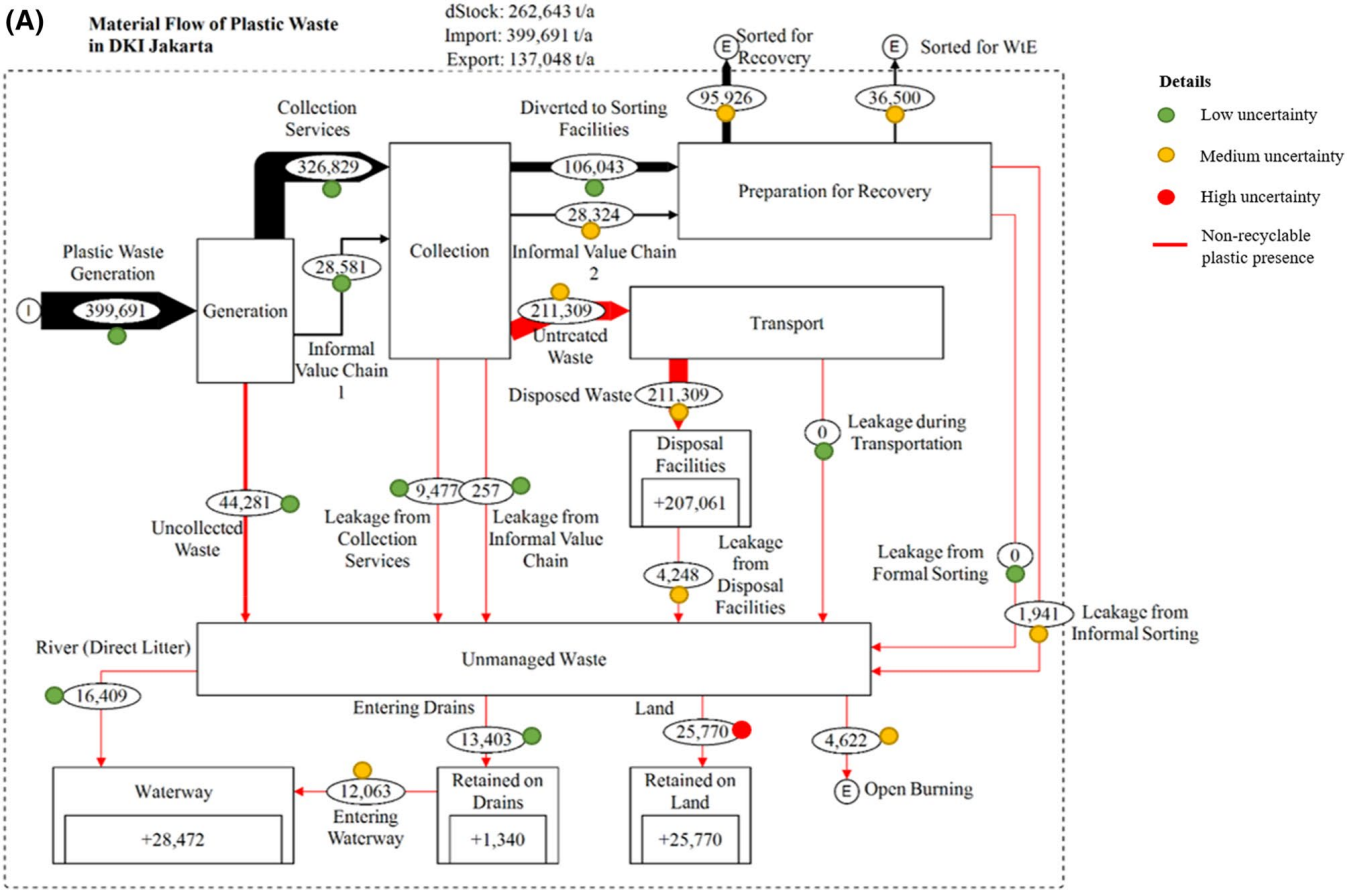

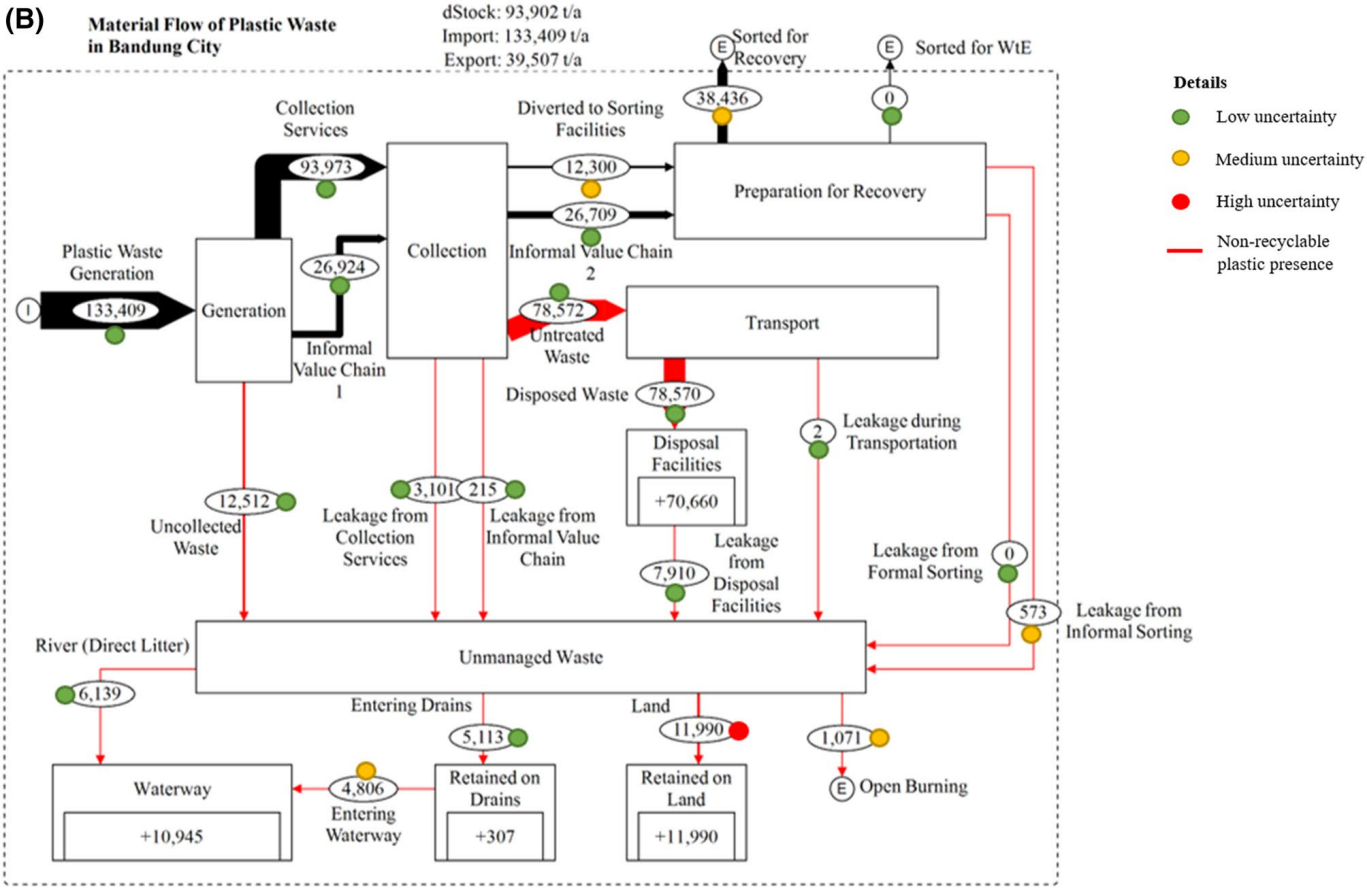
Material flow analysis in (top) DKI Jakarta and (bottom) Bandung City identifying the flow of plastic material from the generation to the different fate based on the leakage.

A material flow analysis was developed to study plastic waste before being categorized as NRPW in Jakarta and Bandung. Results showed that plastic waste generation in Jakarta was 399,691 ton/year from 10,534,339 people^35^, with a 0.7 kg/capita/day municipal solid waste (MSW) generation rate^36^, and 14.85% of the MSW being plastic waste^37^. Approximately 39% of Jakarta's plastic waste composition was PET, and 61% was mixed plastic^37^. Meanwhile, for Bandung with a population of 2,584,252 people^38^, the MSW generation rate and the percentage of plastic waste were 0.63 kg/capita/day^38^ and 22.45%^38^, respectively. This plastic composition consisted of plastic bottles, plastic cups, plastic wraps, multilayered plastic packaging, plastic containers, plastic bags, and diapers. Therefore, plastic waste generation in Bandung was 133,409 ton/year. Figure 1 contains the material flow analysis of plastic for both areas.

In terms of plastic waste collection at the source, 44,281 ton/year (11%) was uncollected in Jakarta, while 326,829 (82%) and 28,581 ton/year (7%) were collected by collection services and the informal value chain, respectively. In Bandung, 12,512 ton/year (10%) of plastic waste was not collected, while 93,973 (70%) and 26,924 (20%) ton/year were collected by collection services and the informal value chain, respectively. The informal value chain is related to informal waste pickers (e.g., scavengers) collecting waste from the streets and disposal sites. With regards to management after collection, collection services diverted waste to the sorting facility at 106,043 and 12,300 ton/year in Jakarta and Bandung, respectively. The higher percentage in Jakarta is supported by waste sorted for energy, with a capacity of approximately 100 ton/day^36^, which also supports the potential for 34% of plastic waste to be recycled as an energy form^39^. Respectively, 95,926 and 36,500 ton/year were sorted from pre-treatment facilities for recycling and energy from waste in Jakarta. Meanwhile, 38,436 ton/year of plastic waste was sorted for recovery (e.g., mechanical recycling) in Bandung, with no waste to energy conversion. Further, a total of 211,309 ton/year of plastic waste was transported by Jakarta’s municipality for final disposal in Bantar Gebang, Bekasi City. In Bandung, 78,570 ton/year of plastic waste was disposed in Sarimukti, West Bandung District.

From both cities, leakage was based on uncollected waste and the residual term of management stages. Jakarta was responsible for 28,472 ton/year of plastic waste entering the waterway across five cities. Whereas, Bandung discharged approximately 10,945 ton/year to the waterway. Most of the plastic waste leaked to the waterway was sourced from direct disposal, which contributes to 57.6% in Jakarta and 56.08% in Bandung, indicating the contribution to litter from human activity, our first phase of investigation^40^. This demonstrates that the main land-based source of plastic debris into the waterway is through direct littering, due to an inadequate waste management system^12,13,41^.

### Quantity and composition of non-recyclable plastic waste leakage

Plastic leakage from uncollected waste was the most significant contributor in both areas, but leakage from other waste management activities was also considered. In Jakarta, 9477 and 257 ton/year leaked from collection services and the informal value chain, respectively. In Bandung, 3101 and 215 ton/year leaked from collection services and the informal value chain, respectively. Apart from collection, disposal facilities in both areas added to the leakage by 4248 ton/year in Jakarta and 7910 ton/year in Bandung. Despite higher amounts of disposed plastic waste in Jakarta than in Bandung, the leakage from disposal facilities was less because we indicated lower leakage potential levels from environmental hazards (e.g., flooding or landslides) and fencing in the Waste Flow Diagram (WFD) tool. For example, we found that treatment and environmental management were adequate in Jakarta’s final disposal and treatment facility, TPST Bantar Gebang^42^. In terms of sorting facilities, formal sorting (e.g., waste banks) in both Jakarta and Bandung does not contribute to plastic leakage since the waste banks sort out valuable plastics at the source. As a result, the amount of rejection from waste banks is very low. On the contrary, 1941 and 573 ton/year of plastic waste was leaked from informal sorting in Jakarta and Bandung, respectively.

When the plastic waste entered the environment, 4622 ton/year was burnt and 25,770 ton/year was retained on land, while 13,403 and 16,409 ton/year entered Jakarta’s river and drainage systems, respectively. Further, 1340 ton/year was retained in the drainage systems, while the remaining 12,063 ton/year was discharged into the waterway. Hence, a total of 28,472 ton/year of plastic waste was leaked into the waterway in Jakarta. When disposable diapers were taken into account, the number increased to 40,880 ton/year since they are responsible for approximately 30%^43^ of the total waterway waste leakage composition in Jakarta. We also included disposable diapers in this study since they are considered an NRPW. In Bandung, 1071 ton/year of plastic waste was leaked into the air through open burning, 11,990 ton/year was retained on land, and 5113 ton/year entered storm drains. Moreover, 6139 ton/year was littered directly into the river. Therefore, 10,945 ton/year of plastic waste was leaked into the waterway in Bandung. Further, the ratio of plastic waste littering per person in Bandung and Jakarta are 3.88 kg/year and 4.24 kg/year, respectively.

**Figure 2:**
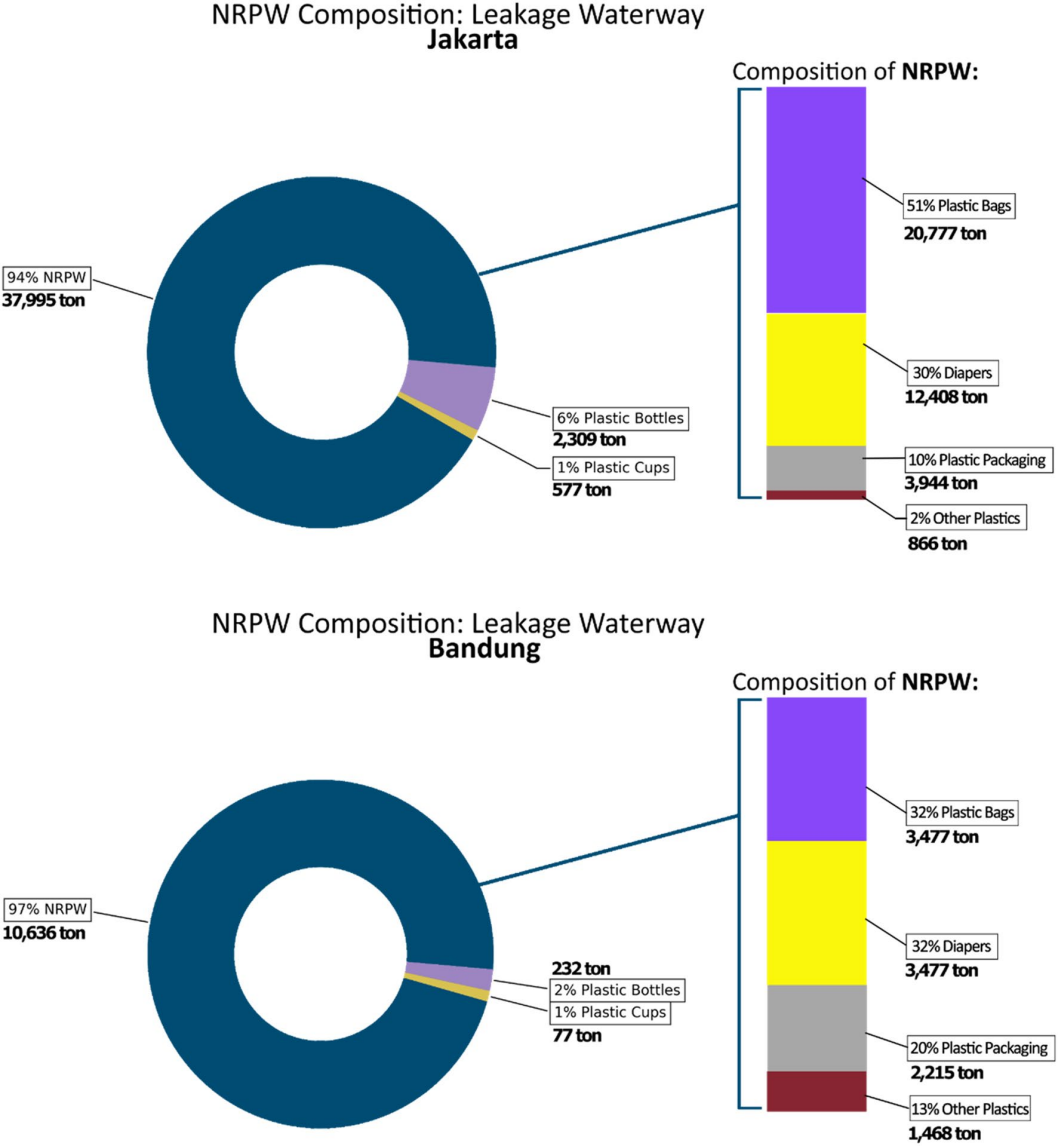
Plastic waste composition in (top) DKI Jakarta and (bottom) Bandung City for the composition of non-recyclable plastic waste (NRPW) components included in both study area.

From the total plastic waste leakage into the waterway, we estimated that the amount of NRPW in Jakarta and Bandung was 37,995 (9.51%) and 10,636 ton/year (7.97%), respectively. Plastic bottles and cups were not included as NRPW due to the availability of recycling facilities and their relatively higher economic value, which is different from that of disposable diapers, multilayered plastic packaging, plastic bags, and other plastic. In Jakarta, plastic bags were identified as the most common NRPW in the waterway, contributing to 20,777 ton/year, owing to the fact that waste is frequently disposed of inside plastic bags. This was followed by 12,408 ton/year of disposable diapers, 3944 ton/year of plastic packaging, and 866 ton/year of other plastic. Meanwhile, in Bandung, plastic bags and diapers were discovered as the most common NRPW in the waterway, contributing the same amount of 3477 ton/year, followed by 2215 ton/year of plastic packaging and 1468 ton/year of other plastic. Based on our model, the composition of NRPW in the waterway for both Jakarta and Bandung is shown in Fig. 2.

### Potential leakage hotspot identification

**Figure 3:**
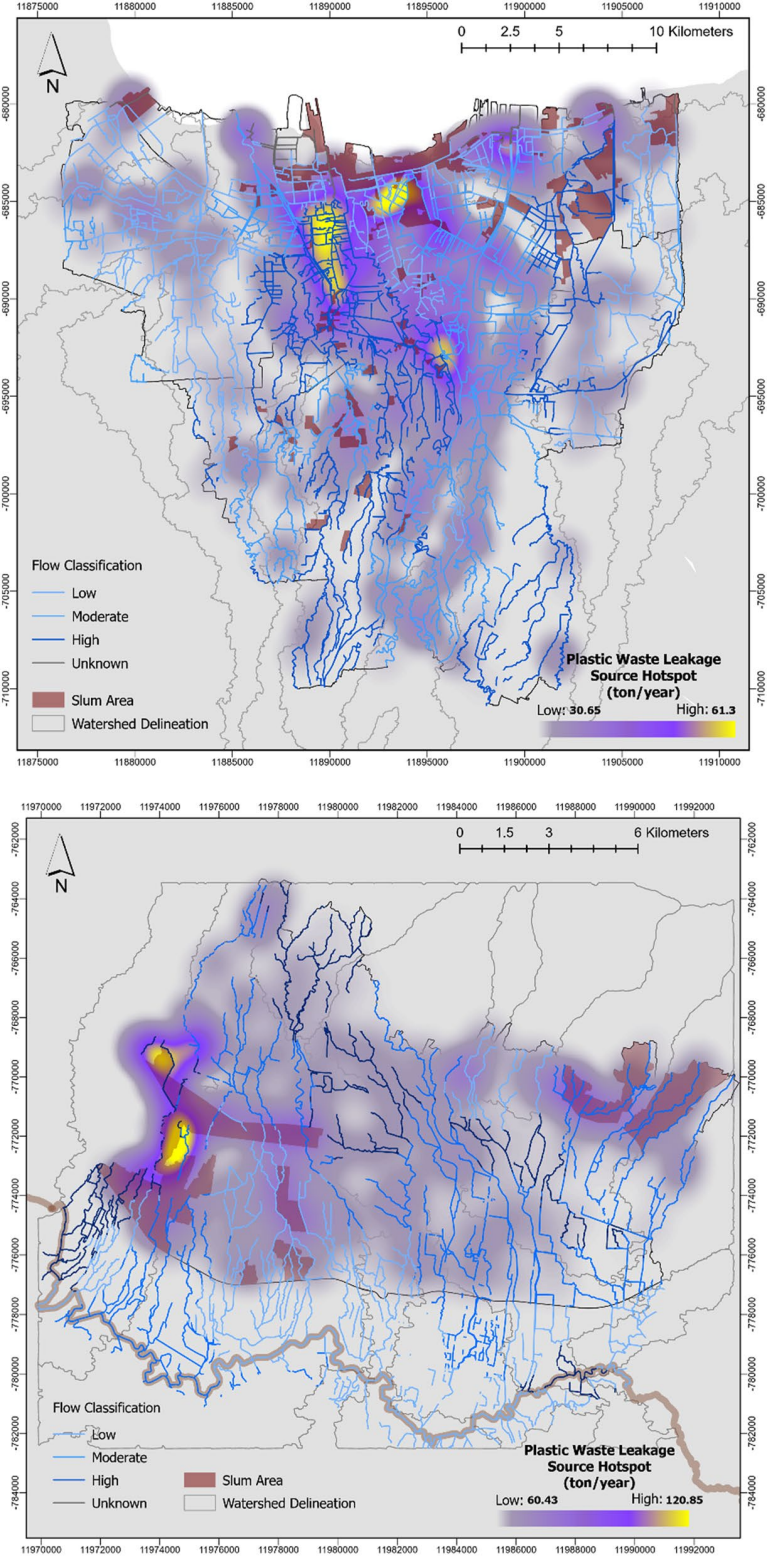
Plastic leakage pathway model in (top) DKI Jakarta and (bottom) Bandung City based on the hotpot from treatment residues.

With the waste generation from the overall population in each city, Jakarta discharged 37,995 ton/year and Bandung discharged 10,636 ton/year to the waterway. From Fig. 3 results show that over 225 locations of hotspots came from waste management facilities in Jakarta, indicating that these facilities are responsible for 24.3% of leakage contributing to the river pathway. From the facility assessment methods and their proximity to the river pathway, spatial distribution indicated that 39.12% of the facilities are in the high-zone proximity (0–100 m) and 7% of the facilities have full potential to cause leakage on an annual basis. Depending on the zonal coverage, most facility leakage was from the *Dipo* types (community level transfer stations). The second greatest contributor was the *TPS 3R* (3R station for material recovery)*,* which exceeded 61.3 ton/year, indicating that the *TPS 3R* concept needs to be improved in Jakarta^44^. Meanwhile, we assumed that the waste bank management was effective, with zero leakage, based on a calculation and assessment through the spatial approach. This implies that collection at the source and current management practices are a positive prospect waste management in Jakarta^45^.

Another source of leakage in Jakarta is from plastic waste generation in residential areas, which contributed to 36.76% of the final disposal^46^. Limited to the population settled in the riverbank area, approximately 2,080,076 people (19.7% of Jakarta’s total population) contribute 28,944 ton/year of plastic waste directly at the source. We found that the potential for direct disposal was due to the cramped population at the wards in slum areas categorized by a population exceeding 87,696 (or 42% of the population settled on the proximity of the river pathway) in between the 0–300 m buffer area from the river centroid across the city.

In Bandung, 122 waste management facilities were contributing to the leakage. Based on the same method for residual leakage, we found that 25 facilities have high leakage probability, in which 14 of the facilities are located close to the riverbank (100-m proximity). Although the leakage hotspots in Bandung are half of that of Jakarta, the minimum leakage exceeds 120.85 ton/year (Jakarta is 61.3 ton/year). In short, Bandung is comparable with Jakarta in terms of waterway leakage based on proportion, where Jakarta contributed 61.3 ton/year from 24.3% possible sources, and Bandung contributed 120.85 ton/year from 20.5% of possible sources of leakage. Therefore, the mobilization of the leakage pathway needs to be concisely constructed.

Overlaid with topographic and hydrological conditions, the morphometric parameters indicated that 31.7% and 18.3% of the waterways in Jakarta and Bandung, respectively, have considerably high plastic leakage. Bandung has higher differences in elevation on the highland area and considering that water flow is likely high in the mountainous areas^47^, there is a higher chance of the plastic waste leakage to shift and accumulate in other areas. Conversely, Jakarta is considerably flat and located in a coastal area; therefore, causing a higher chance of access and leakage accumulation in multiple mainstream waterways.

### Plastic leakage pathway and seasonal changes

**Figure 4:**
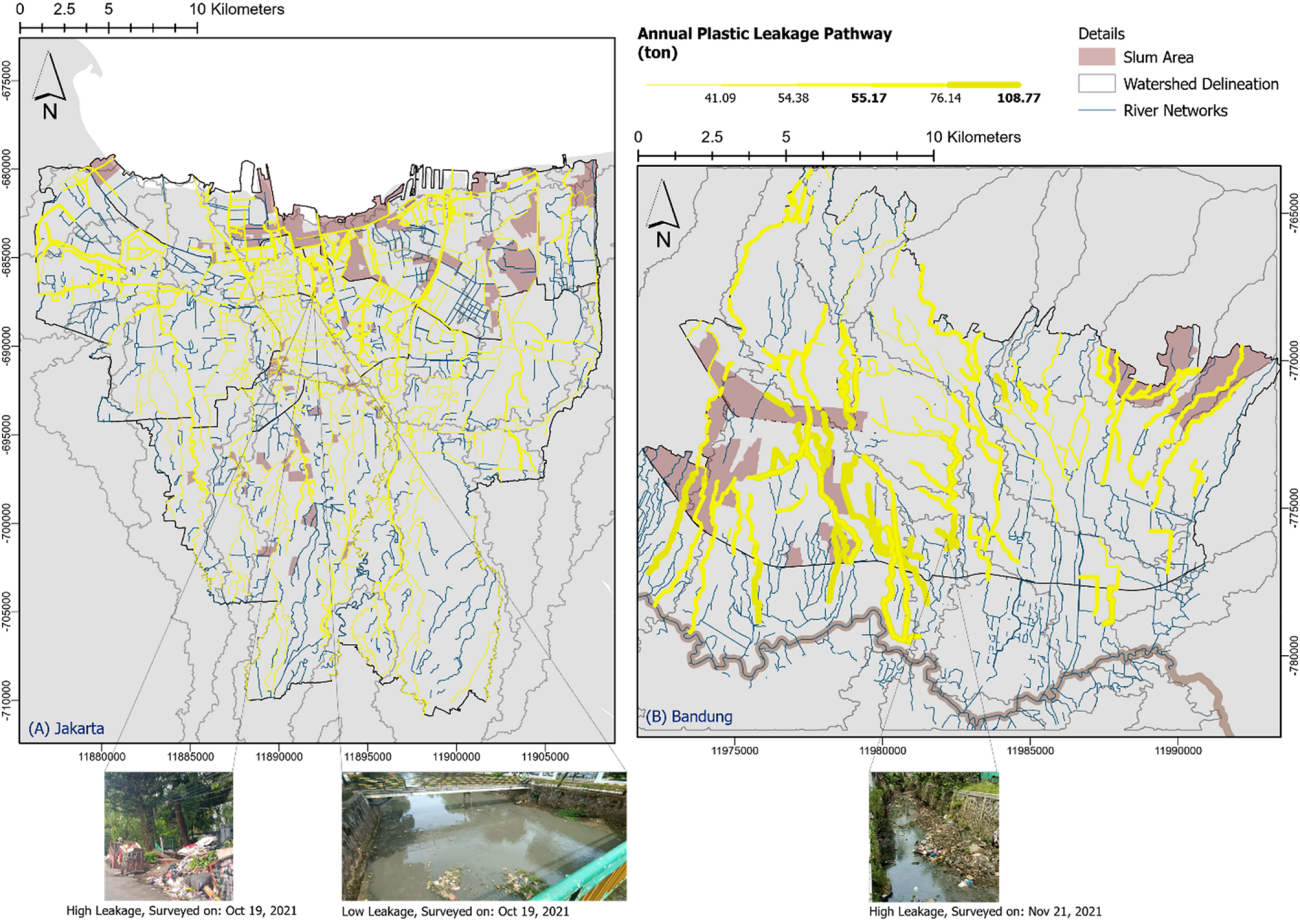
Plastic leakage pathway model in DKI Jakarta and Bandung City based on the accumulation input to the waterway.

Based on the river network distribution, Jakarta is composed of 7 mainstreams that flow directly to the ocean from Jakarta Bay. As shown in Fig. 4, Jakarta obtains the leakage distribution more in the transboundary rivers (western part directly adjacent with Tangerang and eastern part with Bekasi), where potentially 442.58 ton/year was added to the Mookervart River in West Jakarta and 165.51 ton/year to the Cakung River adjacent with Bekasi in North Jakarta.

According to the assessment of the slum area by Statistics Indonesia (BPS) and Indonesia National Slum Upgrading Project (KOTAKU)^48^, there are sparsely dense slum communities in Northern area of Jakarta. The slum area distribution is related to the leakage pathway since there is more plastic waste leakage near the slum-graded wards. We found higher leakage in the streams of the Tanjung Priok port area to be approximately 507.56 ton/year. This provides more evidence that high intensity land and sea-based activity contributes to plastic releases on an annual basis, similar to the results of Jambeck’s 2015 study^49^. Further, direct disposal and insufficient waste management in developing countries is supported by the evidence from the slum area living by the river with a high population density considered in this study^50,51^ and slum area with higher activity and a high leakage load in the northeast part of Jakarta^52^. Additionally, direct disposal also occurs due to unrecorded and unidentified illegal dumping, as reported by Verster and Bouwman in an African case study^53^, which is prevalent in low-managed areas, especially in areas categorized as slums.

The plastic leakage pathway in Bandung (Fig. 4) shows concentrated leakage in the southern part of the city adjacent to Bandung Regency, which leads to the mainstream of Citarum River (represented by dark-colored delineation in Fig. 4). As seen in Fig. 4, the leakage pathway is directed towards the mainstream and obtains a higher amount of input near the tributaries of the Citarum River. As verification of the leakage, we found highly concentrated input to the Cikapundung Kolot waterway from a field survey conducted in November 2021. Correlating with the classified river flow in Fig. 3, the continuous low flow in directions toward the Citarum suggests how a lower flow led to higher accumulation, resulting in high input in the waterway. Thus, around 108.77 ton/year of plastic waste was predicted to be added to the Citarum as the mainstream.

In both Jakarta and Bandung, the condition of accumulation provided by runoff correlated with the rainfall rate (see Supplementary Materials Data S2). We found that rainfall and flash flood identification (from morphometric analysis and peak runoff correlation) corresponds to where most of the mainstreams respond highly to the leakage. Contextualizing the data from satellite-based precipitation^54,55^ and national weather station observations^56^, the peak rainfall in both cities is high from November to May (see Supplementary Materials Data S2 Fig. S3), indicating the possibility of flash floods and fluctuations in plastic leakage to the river pathway.

**Figure 5:**
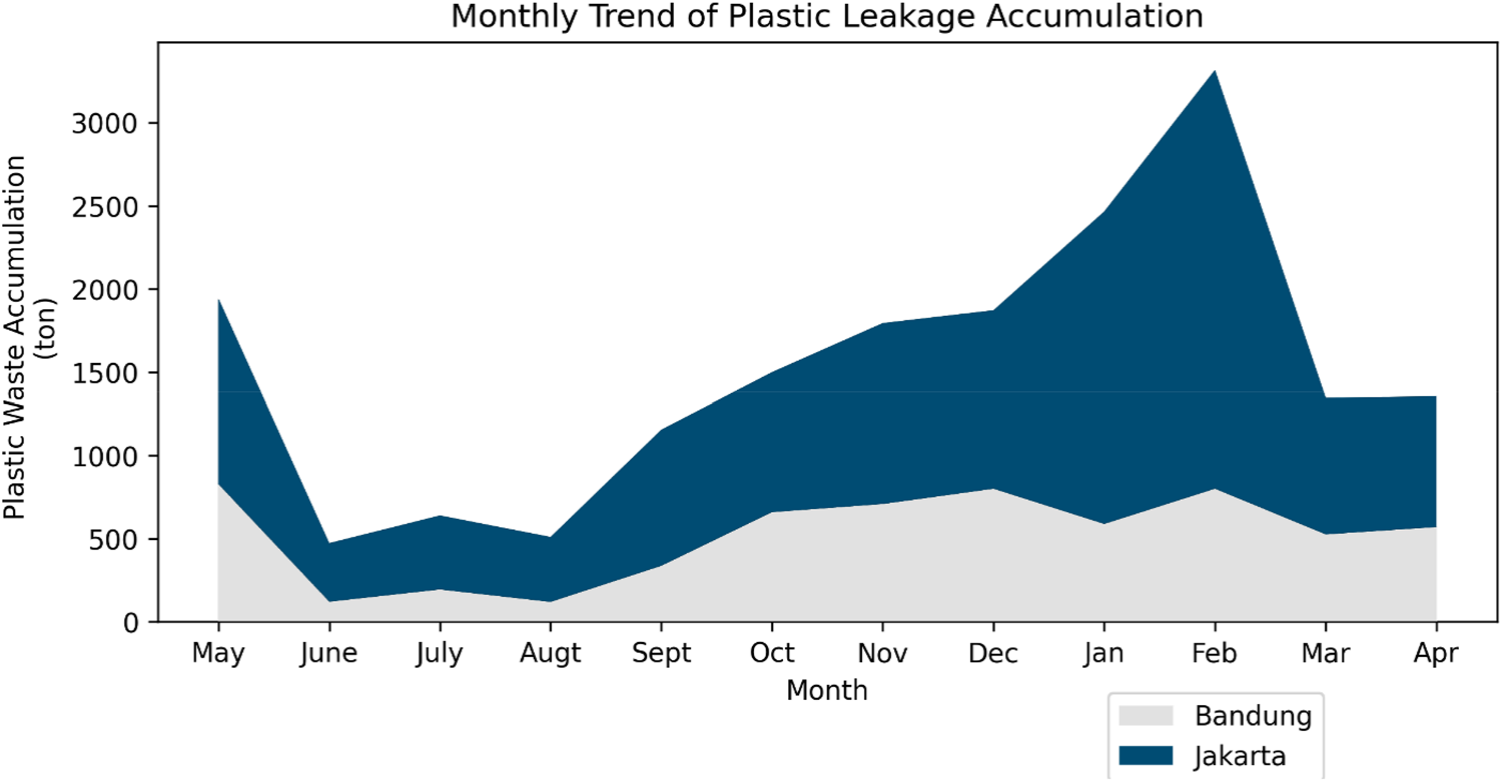
Monthly plastic leakage accumulation in DKI Jakarta and Bandung City modelled for year 2020 to 2021.

When comparing the high rainfall rates in November to May to high pathway leakage input, leakage in Jakarta was exceedingly high in February (Fig. 5). Both cities were high in the transitional period in May where potentially 826.52–1114.82 ton of plastic waste is discarded. We predict that higher rainfall rates, which cause flash floods, correlates with the direct disposal tendency of residents in the riverbank area with lowly managed waste management systems.

Since the peak of leakage accumulation in February in Jakarta exceeded 2,516.44 ton, we investigated the risks from higher rainfall rates (604.4 mm based on Meteorology, Climatology, and Geophysical Agency (BMKG) Kemayoran Meteorological Station^56^). Accordingly, we found that the historical flooding events peaked during the rainy seasons in Jakarta. For a long period of time, historical floods have occurred in Jakarta in February, and 1245 locations have been identified as flooding hotspots from BPBD Jakarta^57^. It is also implied that direct disposal is higher during the rainy season in the riverbank area, which affects significant runoff in the waterway causing a flood. Based on the research from the same location in Jakarta during 2015–2016^58^, the tendency of direct disposal was increasingly high because the high flow removed the waste from the residential area faster. In short, higher rainfall is a complex issue in locations where there is high waste disposal and prevalent flash floods, leading to enormous amounts of plastic waste discarded in waterways.

## Discussion

Plastic waste, especially those categorized as non-recyclable plastics in this study, is leaked to the open environment based on several different perspectives and causal relationships. Firstly, the material flow analysis shows the possibility of leakage according to the judgement of the current management system. Each waste management stage showed that waste contribution was due to the low recyclability of the plastic waste. For example, plastic waste from disposable diapers, multilayered plastic packaging, plastic bags, and other plastics (e.g., toys) have no available recycling method in the cities and/or areas surrounding both Jakarta and Bandung. Although the ability to recycle plastic bags and other plastics might exist at a higher capacity at the community level^59–62^, it is not common practice due to the high contamination of the plastic disposed.

Secondly, based on the multi-criteria assessment and proximity analysis, no relocation is needed for the waste management facilities or residents in the riverbank area. Although the leakage term detected was due to residual and direct disposal, a solution-based approach is preferred from the sources. Therefore, to apply bottom-up solutions, it is important to conduct treatment of facilities. Further, the 3R station (TPS 3R) needs considerable improvement. Although it has been noted to be part of waste reduction, maximizing material recovery is scarce as Jakarta deliberates with Jakarta Recycling Center (JRC) management^63^. For example, waste bank does not emit any leakage which improves from the source collection. This can also be addressed through behavioral changes in human to deliberate with proper management for recycling^64^. Due to their success, waste bank distribution planning and improved accessibility are preferred methods to reduce non-recyclable plastic waste.

**Figure 6:**
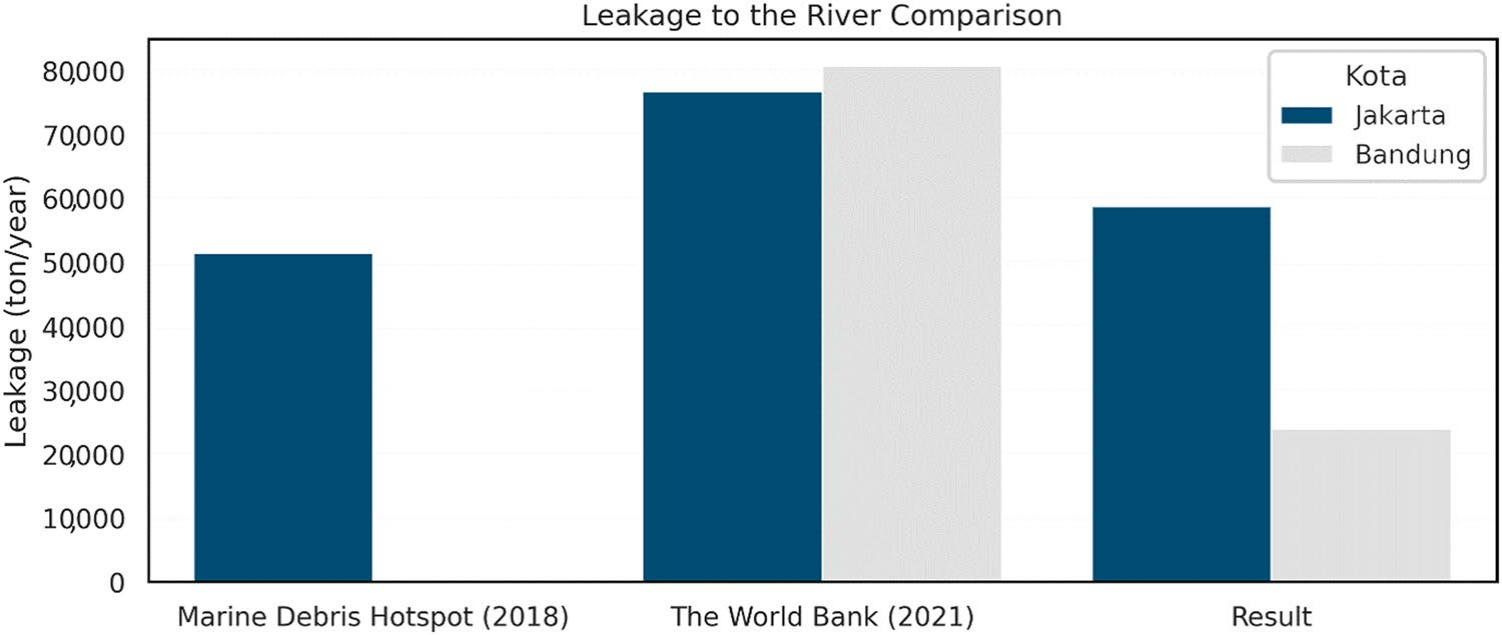
Graphical comparison of plastic leakage to the river pathway through years.

To comprehend the amount of plastic leakage in the waterway, we compared our results to the previous year’s report^43^ and the most recent report^65^ of plastic waste discharge in the waterway. Considering the Marine Debris Hotspot Synthetic Report^43^ defined the problem similar to the terminology of NRPW discharge in non-tidal waterways and the discharge of plastic waste due to mismanaged plastic waste^65^, we reported the proportion of each amount in Fig. 6. Our result values were lower than that of the previous reports^65^, which obtained 76.5% in Jakarta and 29.7% in the Bandung area compared to The World Bank’s result. We then investigated the different approaches towards calculating and classifying the plastic waste in the previous studies, and the World Bank report applied considered overall mismanagement of plastic waste without specifying the plastic types^65^. Therefore, their values were much higher than our results. Additionally, the Marine Debris Hotspot report^43^ showed the ratio of 10:9 differences within 5-year gap of data.

We concluded that the amount of leakage during the rainy season was approximately 785.06–2516.44 ton/month for the five cities in Jakarta and 524.97–826.52 ton/month in the overall area of Bandung City, indicating a substantial effect of flooding. Jakarta had a higher number of flood occurrences due to the lower land level, and extreme rainfall in February caused Jakarta to be more highly prone to flooding during this month. This was supported by stream flow data from over the past 15 years^66–68^, which showed a higher probability of flooding events in February, and morphometric analysis also supported the flash flood occurrence probability^69^. Based on the data from the previous reports, Jakarta should be alerted in February and Bandung in November, as a higher hazard is indicated for this period^54–56^.

Based on the probability of flood periods or higher chances of flash flood due to high rainfall in Jakarta^70^, we verified our evidence, where February was predicted to be a period of concern^71,72^ due to rainfall patterns since 2013. Recorded by the authority of Water Resources Services (DSDA) in Jakarta, from the observing portal called *Pantau Banjir Jakarta*, 12 of 20 floodgates and observation posts recorded hazard alerts due to the water level in February 2021^73^. Rainfall studies showed the flood inundation highly compensates from the higher rainfall rate^74^, which also aggravates the probability of plastic abundance in the waterway, especially in the settlement area^75^. We conclude that there is an alignment between flood occurrence in Jakarta with the amount of overwhelming plastics discarded, with enormous amounts of rainfall. Further, the rainfall rate spikes in Bandung where plastic blocks the flow in Citarum tributaries^76,77^ supports the idea that high plastic accumulation also occurs during the transition from the rainy to dry season.

As a solution, management can be adapted both upstream and downstream for NRPW. We encourage the upstream management to reduce the amount of non-recyclable plastics, such as through design-for-recycling^78^, which encourages producers to re-consider their products to be collectible, detectable, and recyclable^79^. According to an interview with recyclers in Bandung, approximately 30% of plastic waste is rejected from recycling facilities due to bad design. Therefore, there is the opportunity for some producers to make their products recyclable. Another policy approach is the choice editing concept, in which the government and retailers decide on what to (or not to) provide in the market through bans or leveraging sustainable products. In Bandung, we estimate there is approximately 21,895 ton of NRPW that can be avoided through EPR by 2025. In fact, Indonesia has regulated EPR since 2008, yet there are still very few producers who have contributed. To support the implementation of EPR, Indonesia has elaborated on the law with the Ministry of Environment and Forestry (law No. 75/2019) on the roadmap of waste reduction by producers in 2019.

Downstream approach can address the leakage by increasing the management intervention^80^. There are two enabled systems for energy utilization from RDF for industries and upcycling to brick^81^. One of the examples of a community level product is Ecobrick, which proved useful for non-structural purposes^60^ and passed class 3 of National Standards^82^. Considering the urban sprawl in both cities, the location of industrial estate is separate from residential areas in Jakarta and Bandung. Flourishing RDF could be an option for sparsely dense urban cities, which also has been implemented in Central Java^83^.

Our model identified the distribution of locations prone to higher leakage over the years, which can be used to contemplate direct actions in high-risk locations. Yet, robust data of survey location and remote sensing observation are needed to enhance the reliability of the amount of leakage along the plastic value chain. We also recommend designing a direct sampling of the plastic leakage pathway in the river to consolidate the number of leakages, thus considering distribution regardless of the communities. As dynamic changes in the plastic leakage pathway are due to the socio-economic aspects of human activity and management in the area, the solutions need to provide citizen science input.

In this study, we showed that the presence of non-recyclable plastic waste increases the possibility of leaking into the environment, which can receive more than 100 ton plastic waste annually. We detected that the leakage was mainly located in the slum settlement area and curbside of the city due to natural hazards and the overwhelming consumption stage. Therefore, improving urban planning management and waste collection capacity is the primary strategy to prevent leakage. Ultimately, we emphasize direct river cleanup strategies to remove the existing plastic leakage as mapped in our model, while implementing source segregation through waste bank schemes to reduce the presence of non-recyclable plastic.

## Methods

### Material flow analysis: waste flow diagram

#### Plastic waste data on MSW management

In this study, we included the entire area of Bandung and Jakarta (excluding the Kepulauan Seribu district). Jakarta, the capital, is the largest city in Indonesia, with a total area of 662.33 km^2^^84^. The area is divided into six municipalities, 44 sub-districts, and 267 villages. Bandung is the capital city of West Java province. It is divided into 30 sub-districts covering 151 villages, with a total area of 167.31 km^2^^85^. A set of data regarding waste management was collected and entered in the WFD tool. At first, to estimate the plastic waste generation in both cities, the amount was calculated by multiplying the population number, MSW generation rate, and plastic waste fraction in MSW. Secondly, we entered the number of current waste treatment and disposal, which we divided into plastic waste disposed of in disposal facilities, plastic waste sent to waste-to-energy facilities, plastic waste sorted for recovery from both formal and informal sectors, and plastic waste in the informal service chain. Moreover, we also predicted the amount of waste managed in controlled facilities judging from level of control^86^ of a recovery or disposal facility.

#### Data collection methods

We collected the data needed in this study from primary data such as interviews and questionnaires with waste municipality, waste generators (households and non-households), and waste recyclers by direct encounters or remotely due to the Covid-19 pandemic situation. The sample size of these primary data collection is shown in Supplementary Materials Data S3 Table S6. Meanwhile, secondary data such as reports, published documents, and websites were gathered from local authorities, governmental institutions, and organizations. We used the most updated data (2019–2021) for the calculation and obtained the information needed from other reports to create estimations. To represent data certainty, we classified the data into low, medium, and high uncertainty (see Supplementary Materials Data S3 Table S3).

#### Plastic leakage identification

By using the WFD tool, we could estimate the source and fate of plastic waste leakage^87^. We conducted field observations and interviews within both cities to judge the current situation of waste management infrastructure and practices from different stages, which are waste generation, collection, sorting, transportation, and disposal. These judgments were used to input leakage potential levels (none, low, medium, high, or very high) in the WFD tool (see Supplementary Materials Data S3 Table S4), in which was accompanied with a leakage factor representing the percentage of plastic that could leak into the environment^87^. Furthermore, the fate of plastic waste leakage was assessed into four destinations (see Supplementary Materials Data S3 Table S5), which were burnt, retained on land, storm drains, and waterway^87^. Through field observation and interviews within both cities, we estimated the amount of plastic waste in the environment for each fate. Further, the material flow used in this study was modified from the results of the WFD tool to reflect the presence of non-recyclable plastic in the flow of plastic waste.

#### Non-recyclable plastic waste composition analysis

We used a two-step approach to estimate the composition of NRPW in the waterway. Firstly, the amount of plastic waste leakage in the waterway was taken from the results of material flow in Jakarta and Bandung. Secondly, we identified the non-recyclable plastics for both cities based on gathered information from stakeholders and report findings. From the plastic leakage results of WFD, we distributed the composition of each type of plastic in the waterway leakage using the available report findings, which contained the city’s waste composition findings for Jakarta and average waste composition findings for Bandung.

### Hotspot and leakage source mapping

#### Residue calculation as integration from material flow analysis (MFA)

Each of the identified location points was assessed based on the leakage assessment concept on the WFD tool in MFA methods. The potential zones were assessed using the multi-criteria assessment, which included profound condition of the zone and proximity to the waterway. We divided the condition of the close proximity into three potential zones—low, moderate, and high—where each zone was divided per 100 m away from the riverbank area. We considered that the higher zone had proximity, which also implied the deficit of naturalization of the river, where build-up supposedly settled in the river mouth^88^ bringing more plastic waste into the river^89^. The conditions of the zone were defined as whether the system was applied in the area, zonal assessment of slum area based on the economic condition and urban sprawl^48,90^, capacity to retain and manage the waste generated, and assembled systematic schedule.

#### Vulnerability indexes

The distribution of the exact amount of the leakage in the city-level based on material flow analysis was calculated by computing the vulnerability index. This study mainly focuses on the index of the close proximity and index on the residual–where both main indexes combined to provide the final index of assessment. Index of assessment calculated for each zone (facilities and residential area) was used in the next step as the vulnerability index. Therefore, as hotspots were identified by combining both vulnerability index of the waste management facilities and residential area, we can overlook the potential hotspot at the city level.

### Leakage pathway modeling

#### Morphometric analysis

Morphometric analysis was conducted for the hydrological characterization of the leakage pathway from recurring flash floods. We generated 18 morphometric parameters to deliver the streamflow identification from each watershed towards the peak runoff^69,91^ and implemented each waterway's potential capability to retain the water volume. Focusing on the topographic scale, morphometric parameters were delivered by improvising DEM as the quantitative measurement approach^92^. The elevation model was utilized with 8 m spatial resolution of DEM^93^, which morphometric analysis was performed from its scale, topographic, shape, and drainage network in metrics^69,91^, in which we investigated its impact to approach measurement by weight to emphasize the judgement from scale and topographic as the greater impact in the morphometric analysis^91^ for concluding the flow.

#### Hydrological characterization

To generalize streamflow classification, we used a statistical classification method based on the quantification of each major morphometric parameter. Firstly, we combined the different parameters of each sub-category (scale, topographic, shape and drainage network) by reclassifying into three-class of response of the peak runoff, whether it is positive or negative correlation^91^. Secondly, we generalized the zonal statistics combination to ensure the judgement of the peak runoff from each sub-category was covered for each whole watershed delineated. Subsequently, we defined the three classes of flow characterization based on best-fit class distributions. To ease the judgement to the pathway, we divided the flow into high, moderate, low, and unknown, according to the capability of water retention. Unknown class was identified to improve the unjointed zone from the delineated watershed area based on the well-drainage identification, which is prevalent in the coastal area; thus, Jakarta fell under the unknown area of watershed.

#### Calculation of plastic waste in the waterway

To comprehend the streamflow and hotspot results, we generated the concept by using proximity analysis for integrating the land-based result to the river pathway^94^. To distinguish the quantification, we assumed based on the composition analysis and the possible occurrences of flash flood^69^. As a condition of higher density than water will sink the material in the water, we concluded that 50% of materials were retained if the conditions of the flow were high–which quantify the amount of lower accumulation of plastic leakage to the waterway. For moderate flow, we considered that 75% materials were accumulated to take the best-fit accordance from low and high class of flow. Therefore, distinct amount of leakage in the waterway was defined annually.

#### Monthly forecast

We utilized rainfall rate to outline the trend monthly, which to show how the rainfall event affects the possibility of the leakage along with the possible flash flood occurrence and the actual flood historical records^57,95^. Due to the coverage in the city level, we used the monthly total of rainfall rate based on the satellite^54,55^, which distributed per 11.25 km^2^. Rainfall rate is measured in mm units, ranging from 0 to 900 mm, depending on the season (wet and dry). To determine the impact of the rainfall rate pattern, spatial aspects, and hydrological response, we developed Eq. (1) below.

1 $${PWL}_{Monthly}=\left(\frac{\Sigma PWL}{12}\right)\times \%SP\times \left(\frac{MP}{3}\right)$$

To predict the distribution of plastic leakage per month, we utilized the annual accumulation, rainfall contribution, and hydrological response data, where *%SP* (streamflow percentage) quantified the rainfall rate in each city based on the lowest and peak value. Subsequently, we calculated the weight of each month’s rate. Other parameters such as flood historical records were added to the monthly basis of rainfall and quantified as the preset value. The differences were identified by implying the closest actual conditions. Rainfall data were validated and verified by station records^56^, where Jakarta is covered by two meteorological stations and Bandung with one station. The validation across the monthly basis rainfall rate amplified the similarity pattern (see Supplementary Materials Data S2).

## Supplementary Information

Supplementary Information.

## Data Availability

All of the data analysed in this study can be accessed in the open source and accessible through https://figshare.com/articles/journal_contribution/Non-Recyclable_Plastic_Waste_Leakage/19672704 which contains of the result and GIS format.
