# Supplementary material for: Fate identification and management strategies of non-recyclable plastic waste through the integration of material flow analysis and leakage hotspot modeling
Source: Sci Rep. 2022 Sep 29;12:16298. doi: 10.1038/s41598-022-20594-w (PMC9520964; doi:10.1038/s41598-022-20594-w)
Supplement: Supplementary file 1 — Supplementary Information. [file 41598_2022_20594_MOESM1_ESM.docx]

Supplementary Materials for

**Fate Identification and Management Strategies of Non-Recyclable Plastic Waste through the Integration of Material Flow Analysis and Leakage Hotspot Modeling**

Aprilia Nidia Rinasti^1,2*^, Indradhi Faisal Ibrahim^1^, Kavinda Gunasekara^2^, Thammarat Koottatep^1,3^, Ekbordin Winijkul^1,3^

Correspondence to: [aprilianidia@ait.ac.th](mailto:aprilianidia@ait.ac.th)

**This PDF file includes:**

Figs. S1 to S3

Tables S1 to S6

**Other Supplementary Materials for this manuscript include the following:**

Data S1 – Morphometric Analysis for Hydrological Characterization

Data S2 – Rainfall Rate Processing

Data S3 – Waste Flow Diagram (WFD) Tool

Data S1. Morphometric Analysis

All of the map data in this study is generated using ArcGIS® Pro (Ver. 2.8) desktop software by Esri. The resources of the software are herein the intellectual property of Esri (Copyright © Esri. All rights reserved. For more information about Esri® software, please visit [www.esri.com](https://aitthai.sharepoint.com/sites/NRPWLeakageResearchWorkspace/Shared%20Documents/General/Manuscript/Nature%20Scientific%20Reports_Aug%205/www.esri.com)).

Categories of hydrological response were identified by generating digital elevation model (DEM). Comprehended with the topographical condition, DEM was also identifying the scale of sub-basin coverage and initializing the judgement on one watershed coverage. Here’s how the morphometric parameters used in this study for Jakarta and Bandung, Indonesia.

Fig. S1. Delineated Area of Hydrological Analysis in Each City

We conducted the segmentation of the large-scale basin area of the river into several delineation watersheds based on the identification of a well-drained basin where the minimum of stream level is level 5 [1].


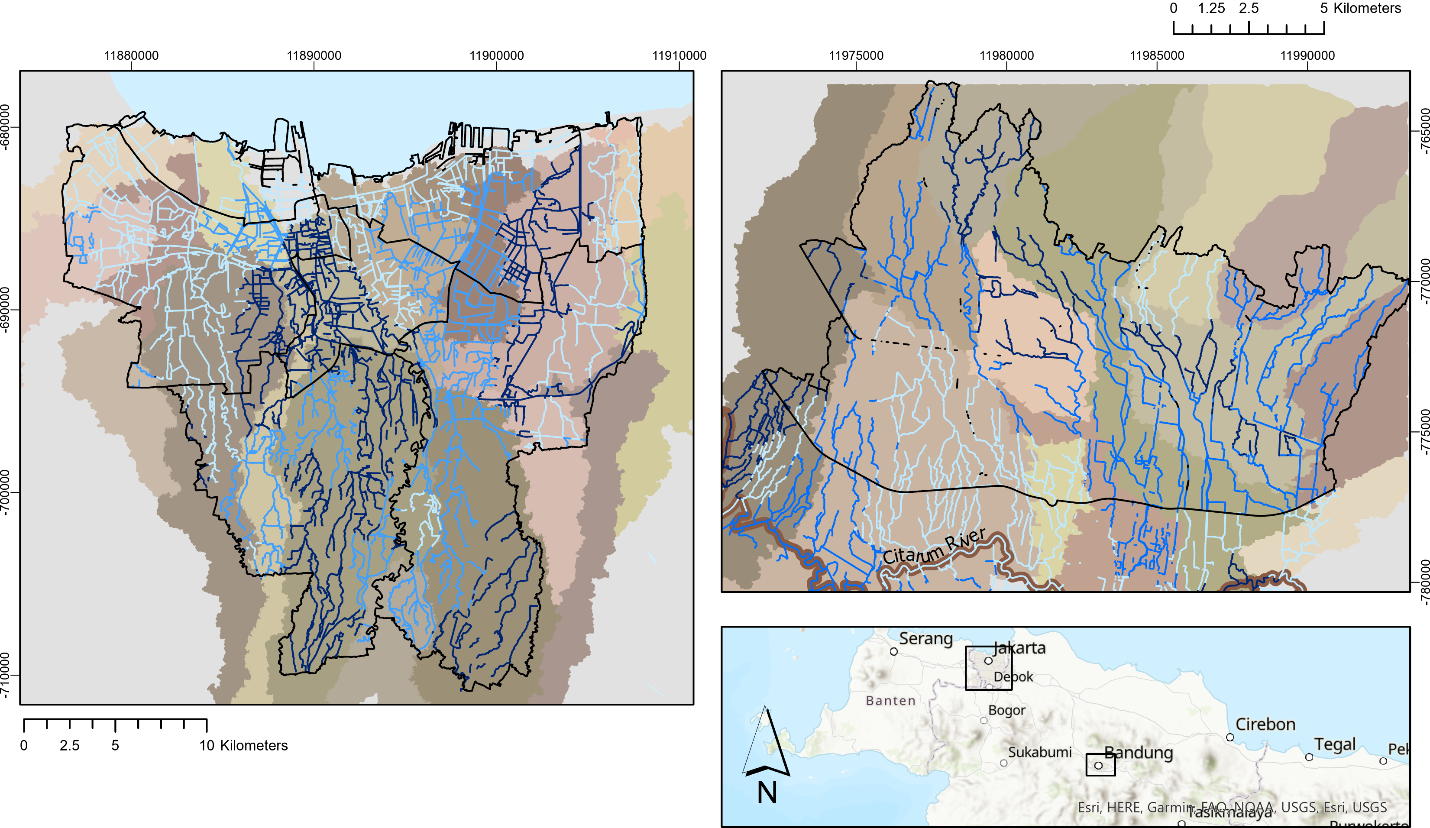


Table S1. Watershed Delineation Result

| **City** | **Total of Watershed** | **Delineated Area**  **(km^2^)** |
| --- | --- | --- |
| Jakarta | 21/34 | 963.16 |
| Bandung | 16/25 | 392.62 |

Table S2. Morphometric Parameters used for the Hydrological Analysis

| **No.** | **Type** | **Parameter** | **Unit** | **Range** | | **Correlation with Peak Runoff [**1**]** |
| --- | --- | --- | --- | --- | --- | --- |
|  |  |  |  | ***Jakarta*** | ***Bandung*** |  |
| 1. | Scale | Area | km^2^ | 4e-05 $-$ 128.95 | 3.57 $-$ 78.58 | Positive |
| 2. |  | Perimeter | km | 0.031 $-$ 159.83 | 13.24 $-$ 84.03 | Positive |
| 3. |  | Length | km | 2.79 $-$ 66.99 | 17.13 $-$ 65.37 | Positive |
| 4. |  | Time Concentration | - | 0.113 $-$ 8176.83 | 0.409 $-$ 636.92 | Negative |
| 5. | Topography | Slope | ^o^ | 0 $-$ 5.9 | 1.81 $-$ 13.81 | Negative |
| 6. |  | Relief Ratio | - | 0 $-$ 7.79 | 0.34 $-$ 22.87 | Positive |
| 7. |  | Mean Elevation | m | 0.38 $-$ 72.95 | 662.71 $-$ 1055.76 | Positive |
| 8. |  | Ruggedness Number | m | 7.9e-04 $-$ 184.58 | 0.0053 $-$ 23.97 | Positive |
| 9. | Shape | Form Factor | - | 2e-08 $-$ 10.41 | 0.0012 $-$ 0.267 | Positive |
| 10. |  | Circularity Ratio | - | 0.037 $-$ 0.572 | 0.0993 $-$ 0.465 | Positive |
| 11. |  | Compactness Ratio | - | 1.32 $-$ 5.21 | 1.46 $-$ 3.17 | Negative |
| 12. |  | Elongation Ratio | - | 1.6e-04 $-$ 3.64 | 0.0398 $-$ 0.583 | Negative |
| 13. | Drainage Network | Stream Order | - | 1 $-$ 5 | 1 – 6 | Positive |
| 14. |  | Stream Number | - | 4 $-$ 169 | 33 $-$ 652 | Positive |
| 15. |  | Stream Length | km | 2.11 $-$ 160.55 | 10.85 $-$ 247.74 | Positive |
| 16. |  | Mainstream Length | km | 0.986 $-$ 66.76 | 2.93 $-$ 71.32 | Positive |
| 17. |  | Drainage Density | km^-1^ | 0.026 $-$ 66,027.6 | 0.138 $-$ 69.39 | Positive |
| 18. |  | Stream Frequency | km^-2^ | 0.061 $-$ 61,567.6 | 0.419 $-$ 182.63 | Positive |
| 19. |  | Texture Ratio | km^-1^ | 0.064 $-$ 245.74 | 0.531 $-$ 49.22 | Positive |

Data S2. Rainfall Rate Processing

Rainfall rate was processed by creating a three-class category of rainfall rate from 4 years period which is shown for both cities in the graph below. To validate the accuracy of satellite-based rainfall records, we conducted a comparison of rainfall rates according to the ground truth station available in both cities.

Fig. S2. Rainfall Trend in 4-year period (A) Jakarta (B) Bandung


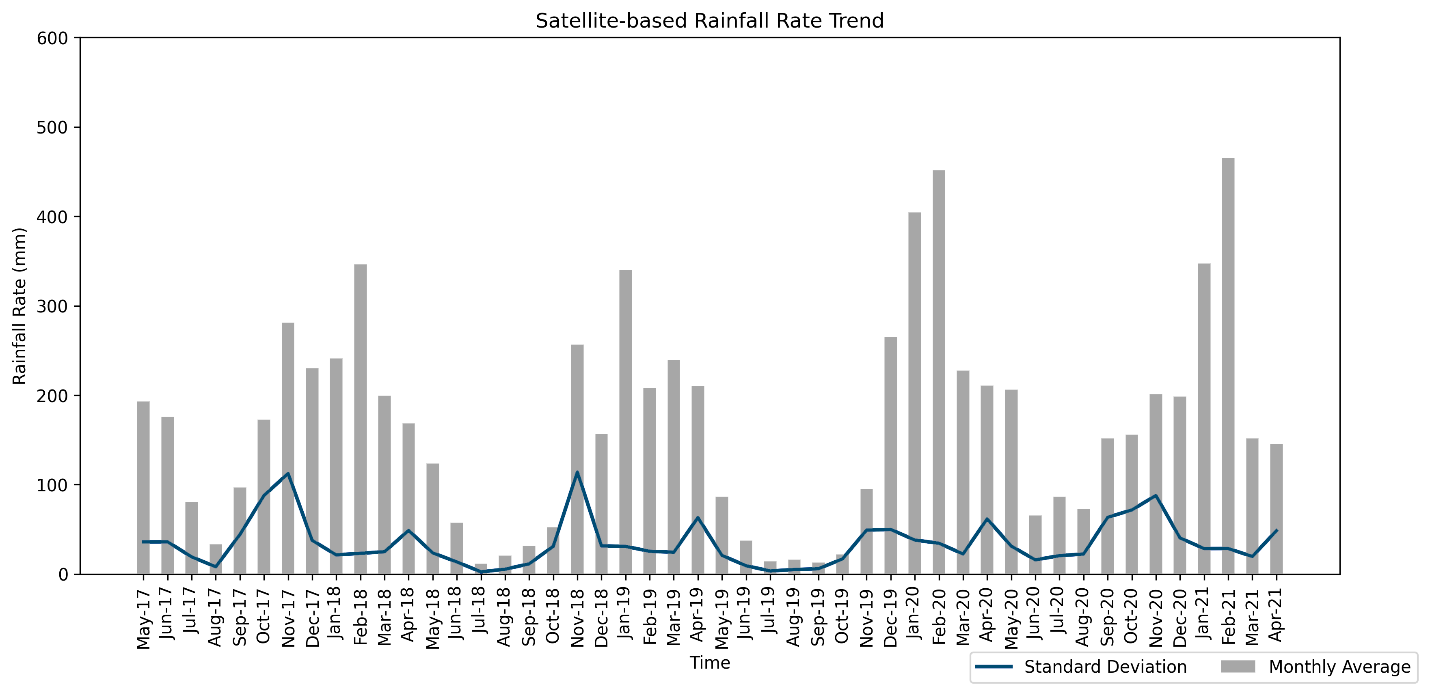


**(A)**


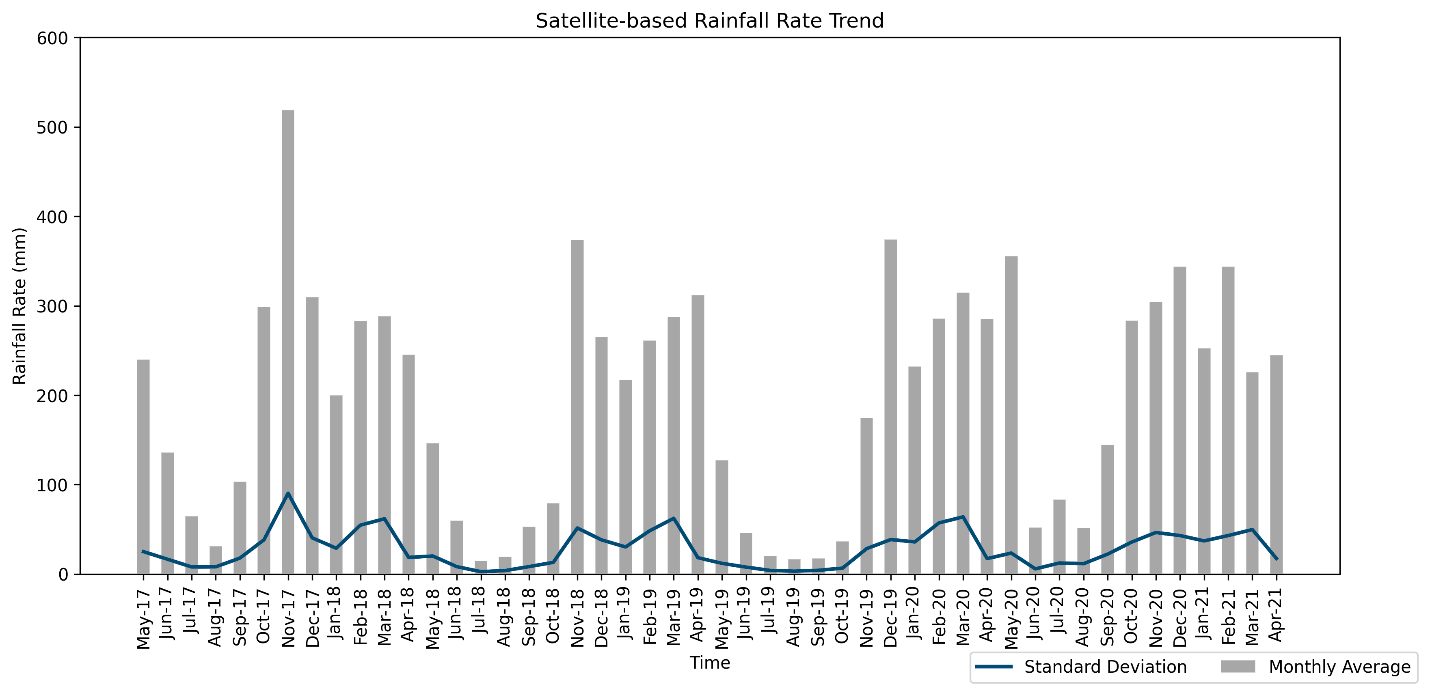


**(B)**

Fig. S3. Rainfall Record Comparisons between BMKG Station and Satellite-based CHIRPS V2.0 (A) Jakarta (B) Bandung


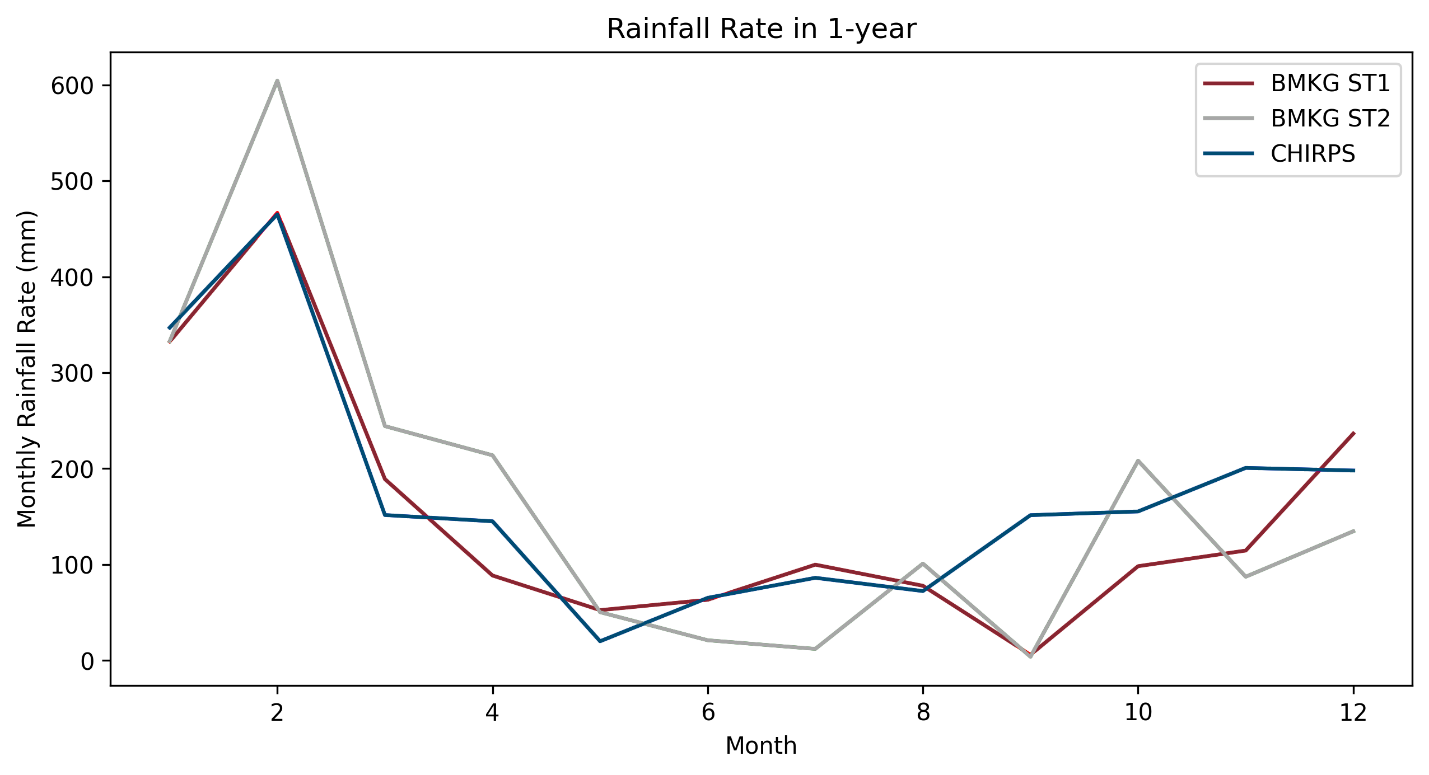


**(A)**


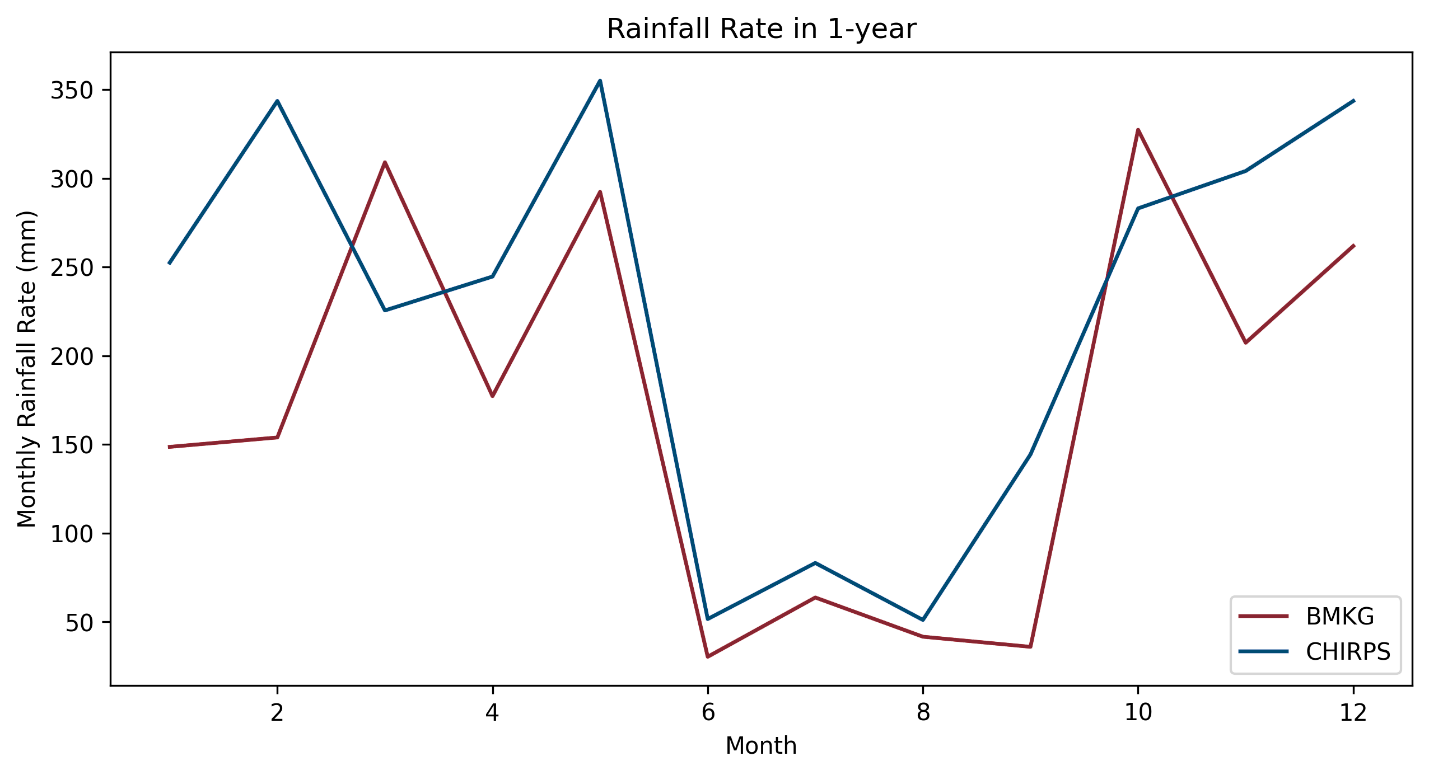


**(B)**

Data S3. Waste Flow Diagram Tool

A set of data regarding waste management was collected to develop the flow of plastic waste using the Waste Flow Diagram (WFD) tool, including the level of data certainty. Field observations were performed directly or through a remote video call with the on-site team in both cities to reflect the current waste management activities such as collection services, informal value chain collection, formal sorting, informal sorting, transportation, and disposal. Further, the fate of pollution was observed such as burnt, retained in the land, retained in drains, and in the water systems. We classified the data certainty into low, medium, and high uncertainty. Low uncertainty comprised data collected directly or gathered from relevant stakeholders, medium uncertainty comprised the estimation based on concrete data, and high uncertainty comprised unknown data, which are calculated based on the principle of mass balance.

Table S3. Waste Flow Diagram Data Inventory (A) Jakarta (B) Bandung

**(A)**

| **No** | **WFD Data** | **Value** | **Estimation/Source** | **Data Certainty** |  |
| --- | --- | --- | --- | --- | --- |
|  |  |  |  |  |  |
| **1** | **Waste generation information** |  |  |  |  |
|  | Population | 10,534,339 persons | *Hasil sensus penduduk 2020 Provinsi DKI Jakarta* (Jakarta Central Bureau of Statistics, 2021) | High |  |
|  | MSW generation per capita | 0.70 kg/capita/day | *Pengelolaan Sampah Jakarta* (Jakarta Environment Agency, 2021) | High |  |
|  | Plastic waste composition | 15% | *Kajian teknis dan hukum revisi perda prov. DKI Jakarta No 03/2013* (Jakarta Government, 2019) | High |  |
|  |  |  |  |  |  |
| **2** | **Waste treatment and disposal** |  |  |  |  |
|  | Disposal facilities | 578.9 ton/day | Based on estimation of total waste disposed multiply by plastic waste fraction | Medium |  |
|  | Waste-to-Energy (WtE) | 100 ton/day | *Pengelolaan Sampah Jakarta* (Jakarta Environment Agency, 2021) | High |  |
|  | Formal sector sorting | 95.6 ton/day | *Rekap Bank Sampah* (Jakarta Environment Agency, 2021) | High |  |
|  | Informal sector sorting | 167.2 ton/day | Mass balance estimation | Medium |  |
|  | % Informal service chain collection | 67% | Website: https://inswa.or.id/jakarta-kewalahan-mengelola-sampah/ | Medium |  |
|  |  |  |  |  |  |
| **3** | **Managed in Controlled facilities** |  |  |  |  |
|  | % of waste sorted for recovery (exclude WtE) | 8.7% | Assumption: Only plastic waste from waste bank is considered to be treated in managed in controlled facilities | Low |  |
|  | % of waste sent for WtE | 9.1% | Assumption: Only from PLTSa Bantargebang data | High |  |
|  | % of waste at disposal facilities | 53% | Interview | Medium |  |
|  |  |  |  |  |  |
| **4** | **Plastic leakage potential levels per leakage influencer** |  |  |  |  |
| *a.* | *Collection services* |  |  |  |  |
|  | Containers/bins | Low | Field Observation | High |  |
|  | Loading method | Medium | Field Observation |  |  |
|  | Primary transportation | High | Field Observation |  |  |
|  | Multiple handling/waste transfer | Medium | Field Observation |  |  |
| *b.* | *Informal value chain collection* |  |  |  |  |
|  | Recyclables extraction method | Medium | Field Observation | High |  |
|  | Transportation method | Medium | Field Observation |  |  |
| *c.* | *Formal sorting* |  |  |  |  |
|  | Reject rate from formal treatment | 10.00% | 9.5% is the amount of plastic residue at waste banks (Putri, A.P. et al, 2018). | High |  |
|  | Rejects from formal treatment | None | Field Observation |  |  |
| *d.* | *Informal sorting* |  |  |  |  |
|  | Reject rate from informal treatment | 14% | Assumption: 14% is the amount of plastic residue at informal sector recycling schemes | Medium |  |
|  | Rejects from informal treatment | Medium | Field Observation |  |  |
| *e.* | *Transportation to disposal facility* |  |  |  |  |
|  | Transport vehicle capacity | Medium | Field Observation | High |  |
|  | Waste containment | Low | Field Observation |  |  |
|  | Transport vehicle cover | Low | Field Observation |  |  |
| *f.* | *Disposal facilities* |  |  |  |  |
|  | Environmental hazard | Low | Interview | Medium |  |
|  | Exposure to weather | Medium | Interview |  |  |
|  | Waste handling | Medium | Field Observation |  |  |
|  | Coverage | Low | Waste is covered typically daily (https://upstdlh.id/tpst/index; Sukwika, T. & Noviana, L., 2020) |  |  |
|  | Burning | High | Interview |  |  |
|  | Fencing | Low | Fence surrounds most of the perimeter and is maintained (https://www.youtube.com/watch?v=bk4yrDBbFgc) |  |  |
| *g.* | *Drains entering waterways* |  |  |  |  |
|  | Rainfall/storm events | Very High | The Koppen Climate Classification subtype for this area is "Af" (https://www.weatherbase.com/weather/weather-summary.php3?s=74769&cityname=Jakarta,+Indonesia) | Medium |  |
|  | Drains clean-up | Low | Interview |  |  |
|  |  |  |  |  |  |
| **5** | **Plastic pollution levels per fate** |  |  |  |  |
| *a.* | *Uncollected plastic waste* |  |  |  |  |
|  | Openly burnt | Low | Field Observation | High |  |
|  | Land | Medium | Field Observation |  |  |
|  | Drains | Medium | Field Observation |  |  |
|  | Water systems | High | Field Observation |  |  |
| *b.* | *Collection and transportation* |  |  |  |  |
|  | Land | Low | Field Observation | High |  |
|  | Drains | Medium | Field Observation |  |  |
|  | Water systems | High | Field Observation |  |  |
| *c.* | *Formal sorting* |  |  |  |  |
|  | Openly burnt | None | Field Observation | High |  |
|  | Land | Very Low | Field Observation |  |  |
|  | Drains | Very Low | Field Observation |  |  |
|  | Water systems | High | Field Observation |  |  |
| *d.* | *Informal sorting* |  |  |  |  |
|  | Openly burnt | Low | Field Observation | Medium |  |
|  | Land | Medium | Field Observation |  |  |
|  | Drains | Low | Field Observation |  |  |
|  | Water systems | High | Field Observation |  |  |
| *e.* | *Disposal facilities* |  |  |  |  |
|  | Land | High | Field Observation | High |  |
|  | Drains | Medium | Field Observation |  |  |
|  | Water systems | Very Low | Field Observation |  |  |

**(B)**

| **No** | **WFD Data** | **Value** | **Estimation/Source** | **Data Certainty** |  |
| --- | --- | --- | --- | --- | --- |
|  |  |  |  |  |  |
| **1** | **Waste generation information** |  |  |  |  |
|  | Population | 2,584,252 persons | Bandung Environment Agency, 2019 | High |  |
|  | MSW generation per capita | 0.70 kg/capita/day | Bandung Environment Agency, 2019 | High |  |
|  | Plastic waste composition | 22% | Bandung Environment Agency, 2019 | High |  |
|  |  |  |  |  |  |
| **2** | **Waste treatment and disposal** |  |  |  |  |
|  | Disposal facilities | 215.3 ton/day | Based on estimation of total waste disposed multiply by plastic waste fraction | High |  |
|  | Waste-to-Energy (WtE) | 0 ton/day | There is no waste to energy recovery in the area yet | High |  |
|  | Formal sector sorting | 20.6 ton/day | *Estimation based on the waste mass balance data* (Bandung Environment Agency, 2019) | Medium |  |
|  | Informal sector sorting | 84.7 ton/day | *Estimation based on the waste mass balance data* (Bandung Environment Agency, 2019) | Medium |  |
|  | % Informal service chain collection | 17% | *Estimation based on the waste mass balance data* (Bandung Environment Agency, 2019) | High |  |
|  |  |  |  |  |  |
| **3** | **Managed in Controlled facilities** |  |  |  |  |
|  | % of waste sorted for recovery (exclude WtE) | 12.9% | *Estimation based on the waste mass balance data* (Bandung Environment Agency, 2019) | High |  |
|  | % of waste sent for WtE | 0.0% | There is no waste to energy recovery in the area yet | High |  |
|  | % of waste at disposal facilities | 87% | *Estimation based on the waste mass balance data* (Bandung Environment Agency, 2019) | High |  |
|  |  |  |  |  |  |
| **4** | **Plastic leakage potential levels per leakage influencer** |  |  |  |  |
| *a.* | *Collection services* |  |  |  |  |
|  | Containers/bins | Medium | Field Observation | High |  |
|  | Loading method | Medium | Field Observation |  |  |
|  | Primary transportation | High | Field Observation |  |  |
|  | Multiple handling/waste transfer | Medium | Field Observation |  |  |
| *b.* | *Informal value chain collection* |  |  |  |  |
|  | Recyclables extraction method | Medium | Field Observation | High |  |
|  | Transportation method | Low | Field Observation |  |  |
| *c.* | *Formal sorting* |  |  |  |  |
|  | Reject rate from formal treatment | 1.00% | Interview | High |  |
|  | Rejects from formal treatment | None | Field Observation |  |  |
| *d.* | *Informal sorting* |  |  |  |  |
|  | Reject rate from informal treatment | 30% | Interview | Medium |  |
|  | Rejects from informal treatment | Medium | Field Observation |  |  |
| *e.* | *Transportation to disposal facility* |  |  |  |  |
|  | Transport vehicle capacity | Medium | Field Observation | High |  |
|  | Waste containment | Low | Field Observation |  |  |
|  | Transport vehicle cover | Medium | Field Observation |  |  |
| *f.* | *Disposal facilities* |  |  |  |  |
|  | Environmental hazard | Medium | Interview | High |  |
|  | Exposure to weather | Medium | Interview |  |  |
|  | Waste handling | Medium | Field Observation |  |  |
|  | Coverage | Medium | Interview |  |  |
|  | Burning | High | Field Observation |  |  |
|  | Fencing | Medium | Field Observation |  |  |
| *g.* | *Drains entering waterways* |  |  |  |  |
|  | Rainfall/storm events | Very High | The Koppen Climate Classification subtype for this area is "Af" (https://www.weatherbase.com/weather/weather-summary.php3?s=18769&cityname=Bandung,+Indonesia) | Medium |  |
|  | Drains clean-up | Medium | Field Observation |  |  |
|  |  |  |  |  |  |
| **5** | **Plastic pollution levels per fate** |  |  |  |  |
| *a.* | *Uncollected plastic waste* |  |  |  |  |
|  | Openly burnt | Low | Field Observation | High |  |
|  | Land | High | Field Observation |  |  |
|  | Drains | Medium | Field Observation |  |  |
|  | Water systems | High | Field Observation |  |  |
| *b.* | *Collection and transportation* |  |  |  |  |
|  | Land | Low | Field Observation | High |  |
|  | Drains | Medium | Field Observation |  |  |
|  | Water systems | High | Field Observation |  |  |
| *c.* | *Formal sorting* |  |  |  |  |
|  | Openly burnt | None | Field Observation | High |  |
|  | Land | Very Low | Field Observation |  |  |
|  | Drains | Very Low | Field Observation |  |  |
|  | Water systems | Very Low | Field Observation |  |  |
| *d.* | *Informal sorting* |  |  |  |  |
|  | Openly burnt | Low | Field Observation | Medium |  |
|  | Land | Medium | Field Observation |  |  |
|  | Drains | Medium | Field Observation |  |  |
|  | Water systems | Low | Field Observation |  |  |
| *e.* | *Disposal facilities* |  |  |  |  |
|  | Land | High | Field Observation | Medium |  |
|  | Drains | High | Field Observation |  |  |
|  | Water systems | High | Field Observation |  |  |

Table S4. Plastic Leakage Potential Levels per Leakage Influencer (A) Jakarta (B) Bandung

**(A)**

| **No** | **Item** | **Leakage Influencers** | **Value** | **Expert Guess Justification** | **Example (Photo/Source)** |
| --- | --- | --- | --- | --- | --- |
| 1.1 | Plastic waste leakage from collection services | Collection containers | Low | - Containers are available in all districts - Open to the environment - Low levels of damage - Waste around the Containers in small quantities - Service occasionally delayed - Waste is disposed of in bags | 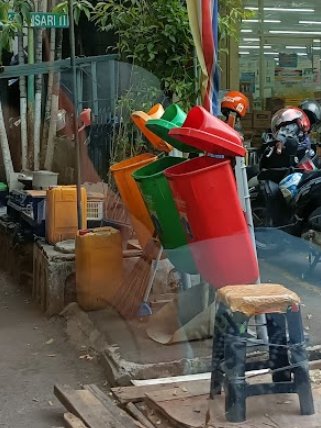 |
| 1.2 |  | Loading method | Medium | - Mostly waste is manually loaded - Containers are generally portable | 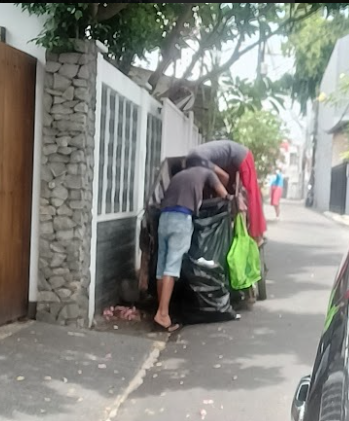 |
| 1.3 |  | Primary transportation | High | - Primary transportation vehicles  mostly small capacity (<5 m3) - Allowing waste to easily escape - Motorcycle & Push/Pull Cart | 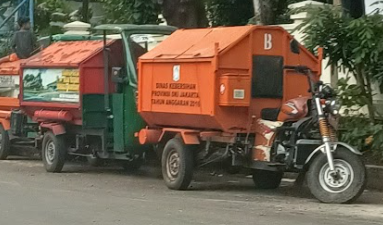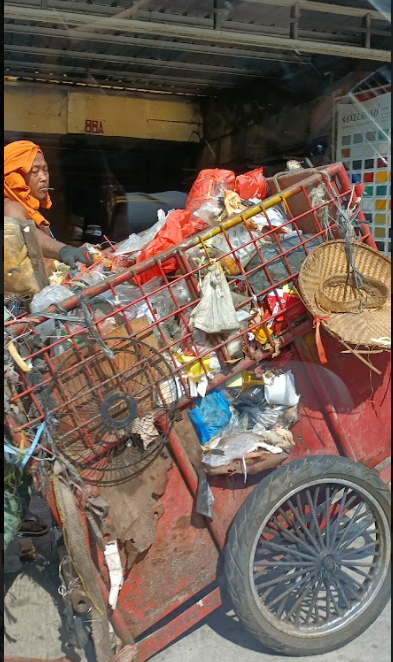 |
| 1.4 |  | Multiple handling/waste transfer | Medium | - Transferred between multiple vehicles/people - Waste typically being loaded onto the ground - Wait times between transfers - Site management is generally adequate | 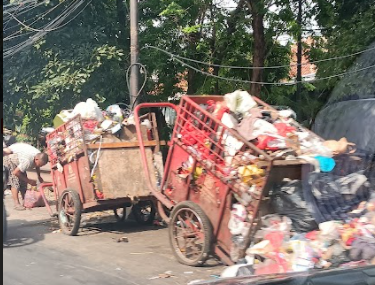 |
| 2.1 | Plastic waste leakage during informal value chain collection | Recyclables extraction method | Medium | - Informal sectors collect during collection - Occasionally discarding unwanted items during  the collection (access to valuable material only) | Assessed during field observation |
| 2.2 |  | Transportation method | Medium | - Transported waste relatively safe (gunny sack) - Over capacity but leakages during transportation is intermittent - Informal sectors tend to  secure their waste (low leakage) | 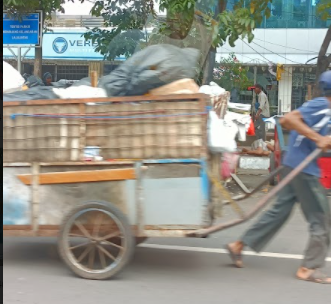 |
| 3.1 | Plastic waste leakage during formal sorting | Formal treatment plastic reject rate | 9.50% | - | Assessed during field observation and literature review [2] |
| 3.2 |  | Disposal of rejects | None | All of the sorting rejects are returned to the formal system | Assessed during field observation and literature review [2] |
| 4.1 | Plastic waste leakage during informal service chain sorting | Informal treatment plastic reject rate | 14% | - | Assessed during field observation and literature review [2] |
| 4.2 |  | Disposal of rejects | Medium | Some sorting rejects are returned to the formal system, however, illegal dumping or burning or rejects is known to occur in some areas. | Assessed during field observation |
| 5.1 | Plastic waste leakage during transportation to disposal | Capacity vs load | Medium | - Most of the truck's load exceeds the capacity | 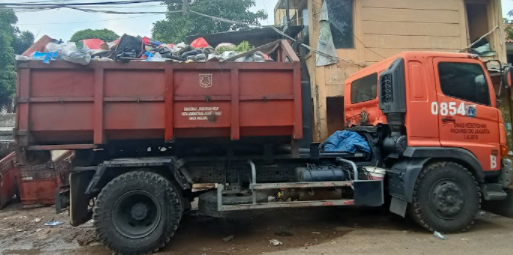 |
| 5.2 |  | Waste containment | Low | - Around half of the generators dispose in bags - Loaders are not practicing the "cherry-pick" method | 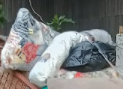 |
| 5.3 |  | Vehicle cover | Low | - Most of the trucks are fully enclosed | 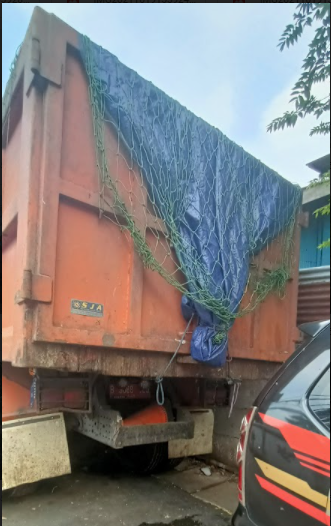 |
| 6.1 | Plastic waste leakage from disposal facilities | Environmental hazards | Low | Interview: Site is located in an area where regular flooding/landslides affect very few parts of the site | Assessed during field observation |
| 6.2 |  | Exposure to weather | Medium | Interview: Sometimes exposed to heavy and persistent winds or runoff in the rainy season. | Assessed during field observation |
| 6.3 |  | Waste handling | Medium | - Field Observation: waste pickers active around discharge zone. - Compaction occurs but with the condition of overload capacity landfill. | Assessed during field observation and literature review [3] [4] |
| 6.4 |  | Coverage | Low | Waste is covered typically daily. | [Assessed during field observation and literature review [5]](https://upstdlh.id/tpst/indexSukwika,%20T.,%20dan%20Noviana,%20L.%20(2020).%20Status%20Keberlanjutan%20Pengelolaan%20Sampah%20Terpadu%20di%20TPST‐Bantargebang%20Bekasi:%20MenggunakanRapfish%20dengan%20R%20Statistik.%20Jurnal%20Ilmu%20Lingkungan,%2018(1),%20107‐118,%20doi:10.14710/jil.18.1.107‐118) |
| 6.5 |  | Burning | High | Burning of waste is rare | Assessed during field observation |
| 6.6 |  | Fencing | Low | Fence surrounds most of the perimeter and is maintained | Assessed during field observation |
| 7.1 | Plastic waste in storm drains entering waterways | Frequency of rainfall/storm events | Very High | The Koppen Climate Classification subtype for  this climate is "Af". (Tropical Rainforest Climate).  [6] | Assessed during field observation |
| 7.2 |  | Drain clean-up | Low | - Majority of storm drains are cleaned regularly - Litter traps are used on a handful of drain outlets and are well maintained. |  |

**(B)**

| **No** | **Item** | **Leakage Influencers** | **Value** | **Expert Guess Justification** | **Example (Photo/Description)** |
| --- | --- | --- | --- | --- | --- |
| 1.1 | Plastic waste leakage from collection services | Collection containers | Medium | - Containers are mostly available - Open to the environment  - Low levels of damage - Waste around the containers - Service occasionally delayed - Waste is disposed of in bags | 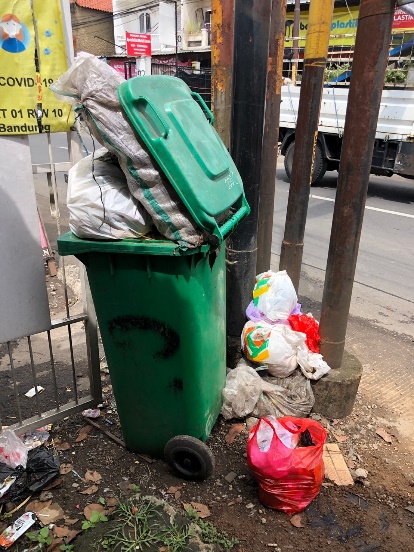 |
| 1.2 |  | Loading method | Medium | - Mostly waste is manually loaded - Containers are generally portable | 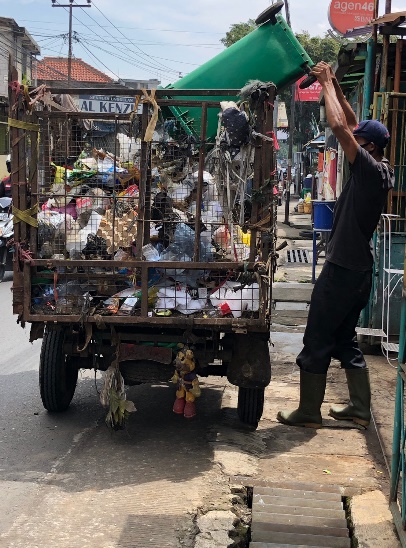 |
| 1.3 |  | Primary transportation | High | - Primary transportation vehicles  mostly small capacity (<5 m3) - Motorcycle & Push/Pull Cart | 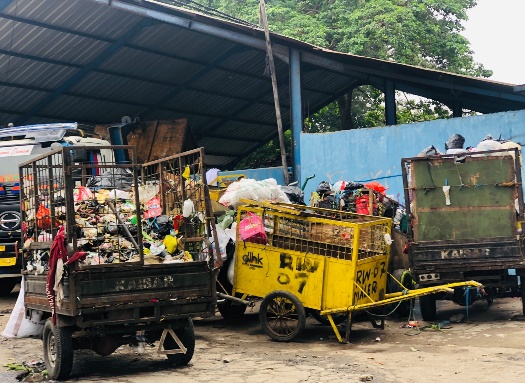 |
| 1.4 |  | Multiple handling/waste transfer | Medium | - Transferred between multiple vehicles/people - Waste typically being loaded onto the ground - Wait times between transfers - Site management is generally adequate | 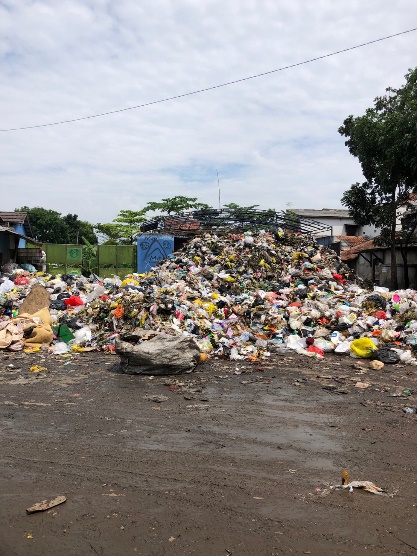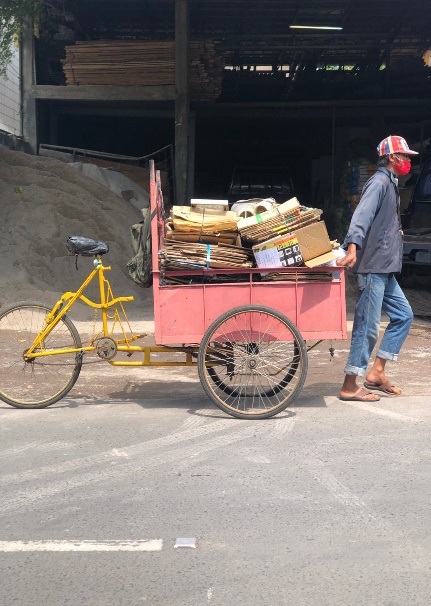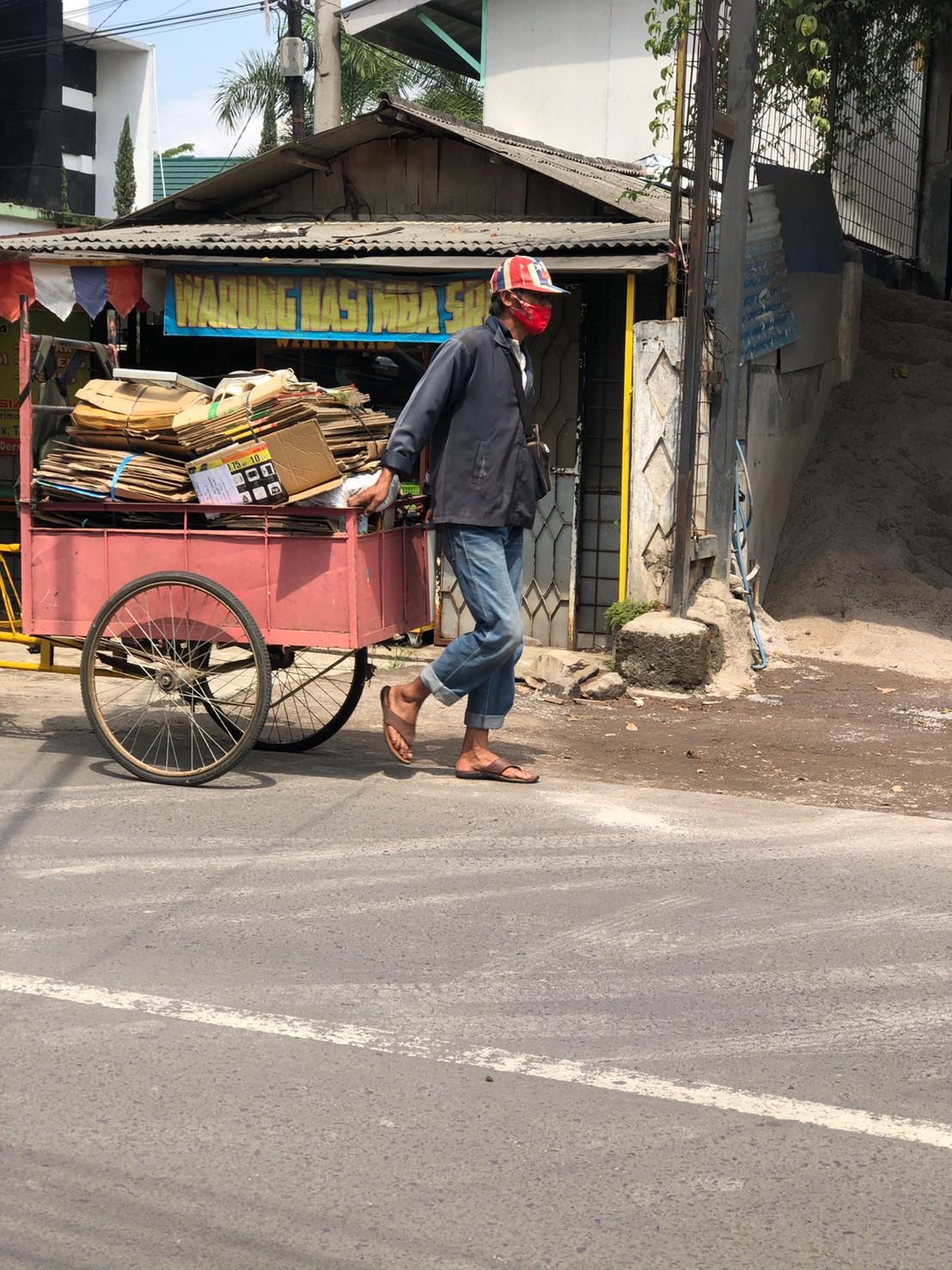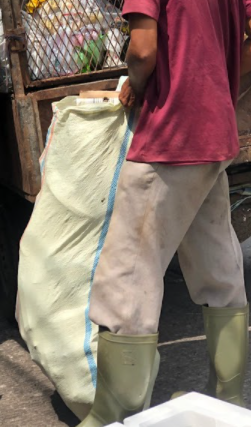 |
| 2.1 | Plastic waste leakage during informal value chain collection | Recyclables extraction method | Medium | - Informal sectors collect during collection - Occasionally discarding unwanted items during  the collection (access to valuable material only) |  |
| 2.2 |  | Transportation method | Low | - Transported waste relatively safe (gunny sack) - Informal sectors tend to  secure their waste (low leakage) |  |
| 3.1 | Plastic waste leakage during formal sorting | Formal treatment plastic reject rate | 0.01 | - Almost no reject.  - Most reject are back to the formal disposal chain. | Assessed during interviews with the waste authority and formal waste bank |
| 3.2 |  | Disposal of rejects | None | - Almost no reject.  - Non-recyclable plastic is rejected since the beginning,  and there is always a buyer for the reject. | Assessed during field observation |
| 4.1 | Plastic waste leakage during informal service chain sorting | Informal treatment plastic reject rate | 0.3 | - Highly contaminated (oil, paint, etc.) | 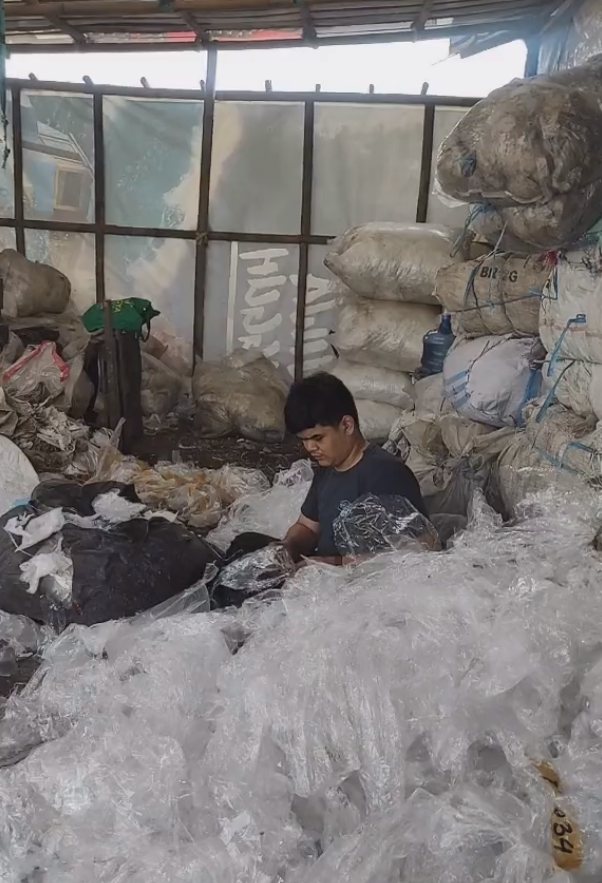 |
| 4.2 |  | Disposal of rejects | Medium | - Most rejects are returned to the formal system - Open dumping of rejects is known to occur | 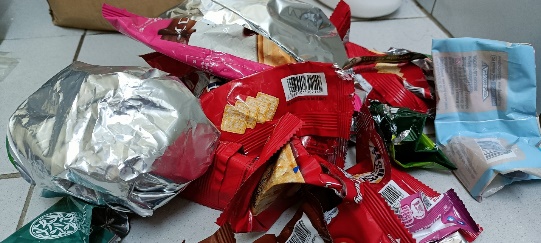 |
| 5.1 | Plastic waste leakage during transportation to disposal | Capacity vs load | Medium | - Around half of the truck's load exceeds the capacity | 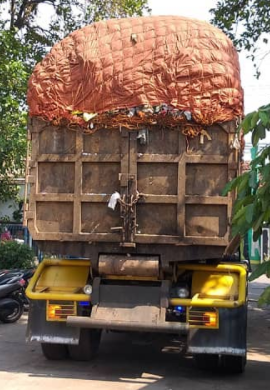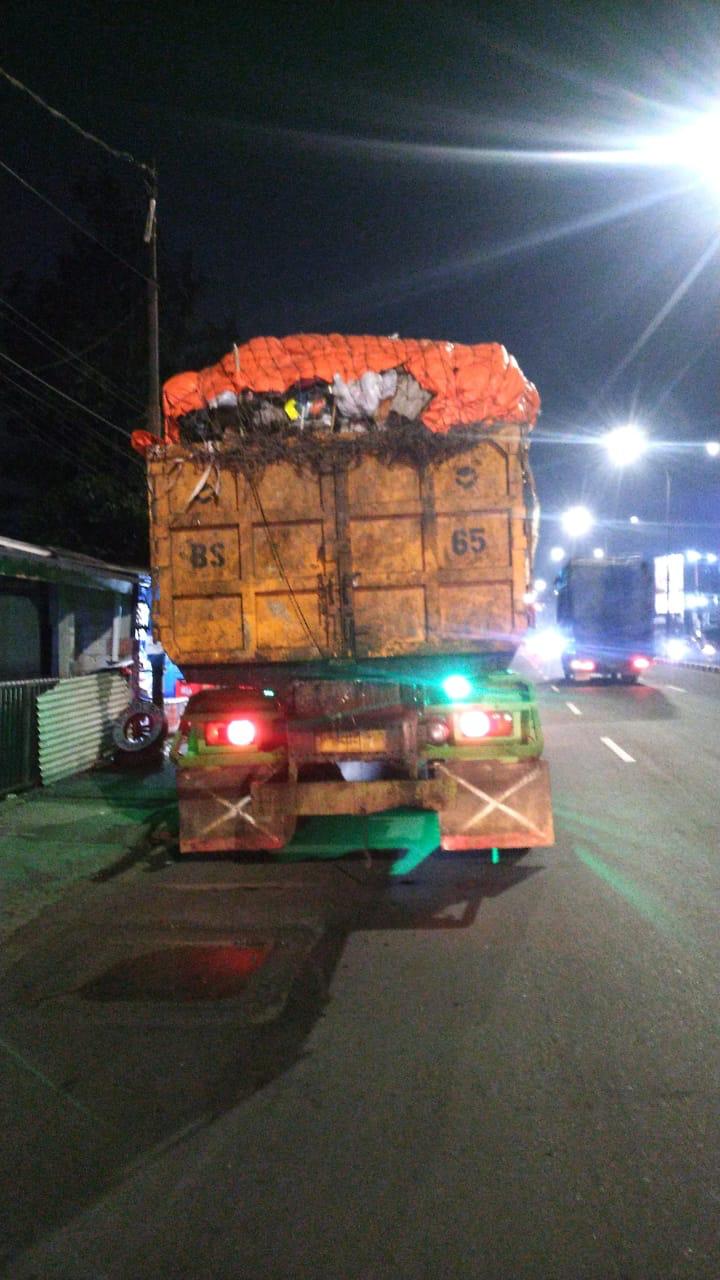 |
| 5.2 |  | Waste containment | Low | - Mostly waste disposed of in bags - Loaders are not practicing the "cherry-pick" method | 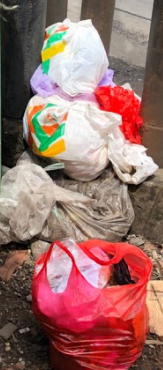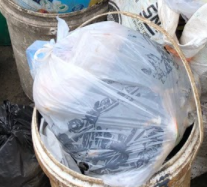 |
| 5.3 |  | Vehicle cover | Medium | - Most of the trucks are fully enclosed | 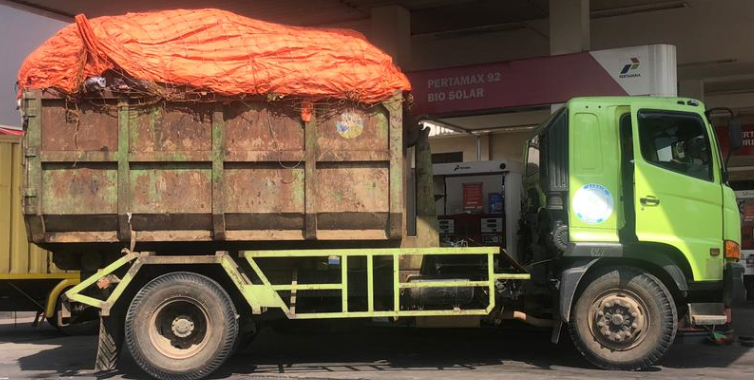 |
| 6.1 | Plastic waste leakage from disposal facilities | Environmental hazards | Medium | Interview: flood and landslide are taking place  during rain due to overcapacity. | Assessed during interview with the waste authority |
| 6.2 |  | Exposure to weather | Medium | Interview: Surface run-off comes  from the higher-level areas. | Assessed during interview with the waste authority |
| 6.3 |  | Waste handling | Medium | - Field Observation: waste pickers active  around discharge zone.  - Compaction occurs but with the  condition of overload capacity landfill.  - Waste is piled above ground with full exposure  to wind, rain, and some surface runoff. | [7]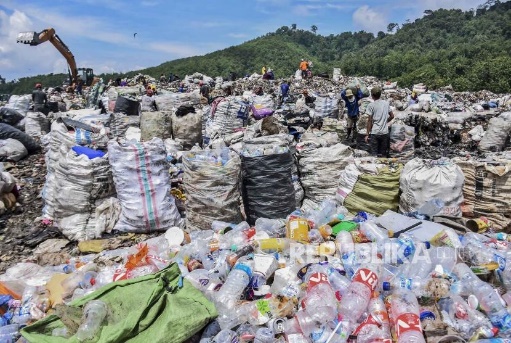 |
| 6.4 |  | Coverage | Medium | Waste is covered typically once per week or daily. | Assessed during field observation |
| 6.5 |  | Burning | High | Burning of waste is rare | Assessed during field observation |
| 6.6 |  | Fencing | Medium | Fence surrounds most of the perimeter  but is broken in several sections | 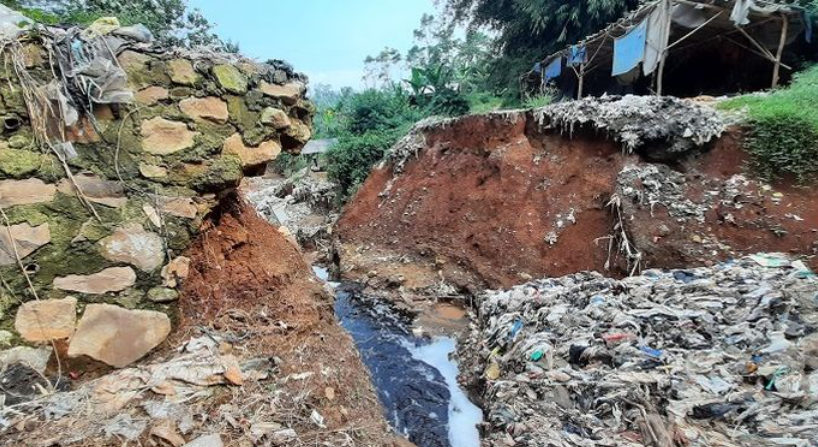[8] |
| 7.1 | Plastic waste in storm drains entering waterways | Frequency of rainfall/storm events | Very High | - The Koppen Climate Classification subtype for  this climate is "Af". (Tropical Rainforest Climate).  [9] | Assessed during field observation |
| 7.2 |  | Drain clean-up | Medium | - Drain clean-up is irregular (based on program),  with this planned to occur before periods of heavy rain  (i.e. wet season if applicable).  - Litter traps are used on a handful of drain  outlets and are well maintained. |  |

Table S5. Plastic Pollution Levels per Fate (A) Jakarta (B) Bandung

**(A)**

| **No** | **Item** | **Level of Fate of Plastic Waste** | **Value** | **Description** | **Example (Photo/Source)** |
| --- | --- | --- | --- | --- | --- |
| 1.1 | Fate of uncollected plastic waste | Level of plastic openly burnt | Low | - There is sporadic evidence of open burning - Not the majority of residents | 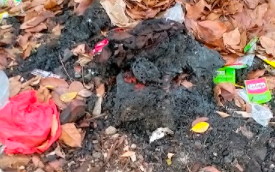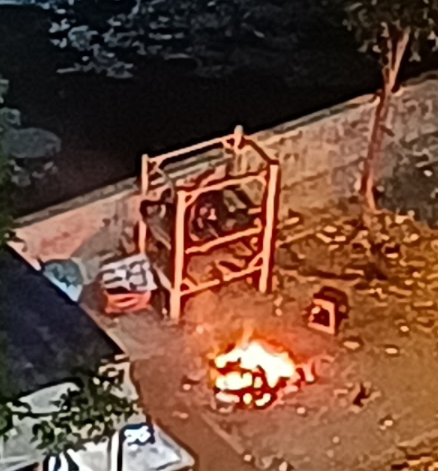 |
| 1.2 |  | Level of direct dumping on land | Medium | - In areas without services, there is evidence that residents may regularly dump to land - There is sporadic evidence and occasional street sweeping occurs | 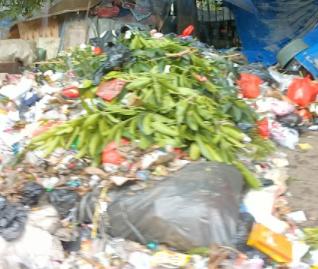 |
| 1.3 |  | Level of direct dumping in drains | Medium | - There is evidence of that residents may dump  their waste but not the majority | 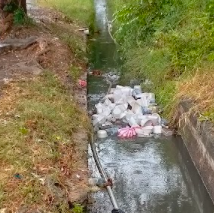 |
| 1.4 |  | Level of direct dumping in water system | High | - In areas without services, majority disposed  of the waste, which they have access to water body | 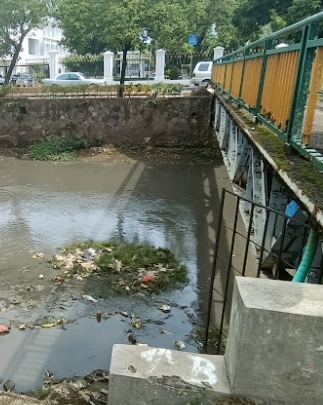 |
| 2.1 | Fate of plastic waste leaked during collection and transportation | Level of plastic to land | Low | - Throughout all the study area, there is evidence  of small quantities of plastic remaining on land | 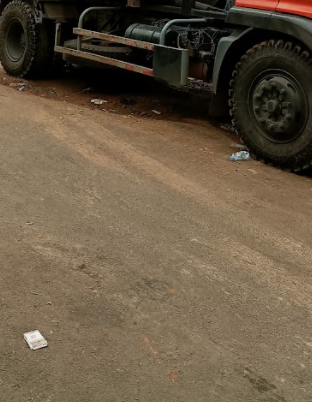 |
| 2.2 |  | Level of plastic to drains | Medium | - Majority of area, there is evidence  of small quantities of plastic leakage - Although a minority of areas show evidence  of large quantities of leakage | 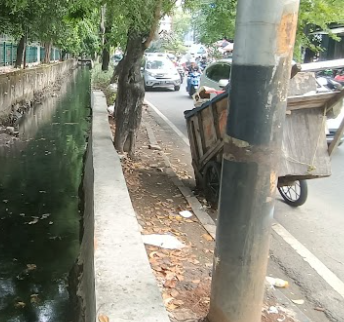 |
| 2.3 |  | Level of plastic to water systems | High | - Majority of the area close proximity (<1 km)  to the water system - Vegetation on the banks of the water systems is sparse throughout large parts of the study area. | 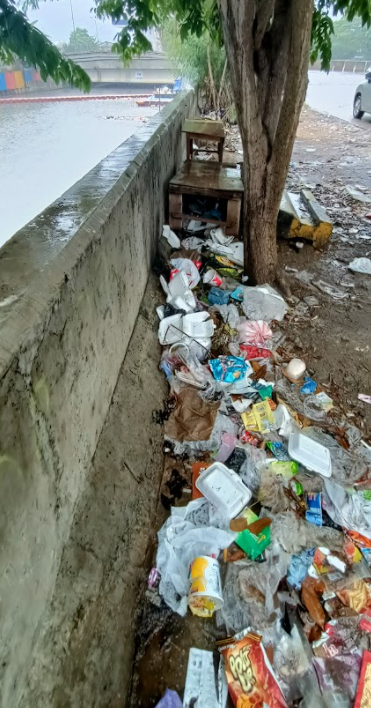 |
| 3.1 | Fate of plastic waste leaked from formal sorting | Level of plastic openly burnt | None | - No evidence of open burning | Assessed during field observation |
| 3.2 |  | Level of direct dumping on land | Very Low | - It is believed to the rare occurrence and unintentionally enter the surrounding land | Assessed during field observation |
| 3.3 |  | Level of direct dumping in drains | Very Low | - It is believed to the rare occurrence and unintentionally enter the drains | Assessed during field observation |
| 3.4 |  | Level of direct dumping to water systems | High | - The majority of sorting facilities are <500 m to water systems and it is believed controlled (981 out of 1149) | 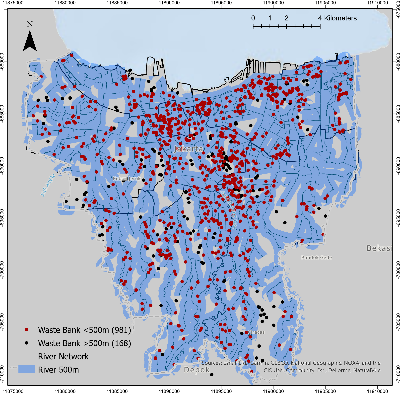 |
| 4.1 | Fate of plastic waste leaked from informal sorting | Level of plastic openly burnt | Low | - There is sporadic evidence, but it is believed to be a rare occurence | Assessed during field observation |
| 4.2 |  | Level of direct dumping on land | Medium | - There is sporadic evidence that sorting facilities may regularly dump their rejects to the surrounding areas, and street sweeping occasionally occurs in the vicinity | Assessed during field observation |
| 4.3 |  | Level of direct dumping in drains | Low | - Minority of sorting facilities may regularly dispose their rejects to drains, and no active cleaning | Assessed during field observation |
| 4.4 |  | Level of direct dumping to water systems | High | - The majority of sorting facilities are in close proximity (<500 m) to water systems of which they have access | Assessed during field observation |
| 5.1 | Fate of plastic waste leaked from disposal facilities | Level of plastic to land | High | - There is evidence of plastic waste remaining on land,  - In fact, the landfill almost reach its capacity | Assessed during field observation |
| 5.2 |  | Level of plastic to drains | Medium | - There is evidence of small quantities of plastic waste entering storm drains although a minority of areas show evidence of large quantities | Assessed during field observation |
| 5.3 |  | Level of plastic to water systems | Very Low | - All the point sources are not in close proximity (>1 km) to water systems. Vegetation on the banks of the water systems is dense | Assessed during field observation |

**(B)**

| **No** | **Item** | **Level of Fate of Plastic Waste** | **Value** | **Description** | **Example (Photo/Source)** |
| --- | --- | --- | --- | --- | --- |
| 1.1 | Fate of uncollected plastic waste | Level of plastic openly burnt | Low | - There is sporadic evidence of open burning - Not the majority of residents | 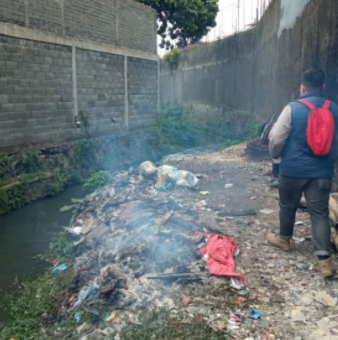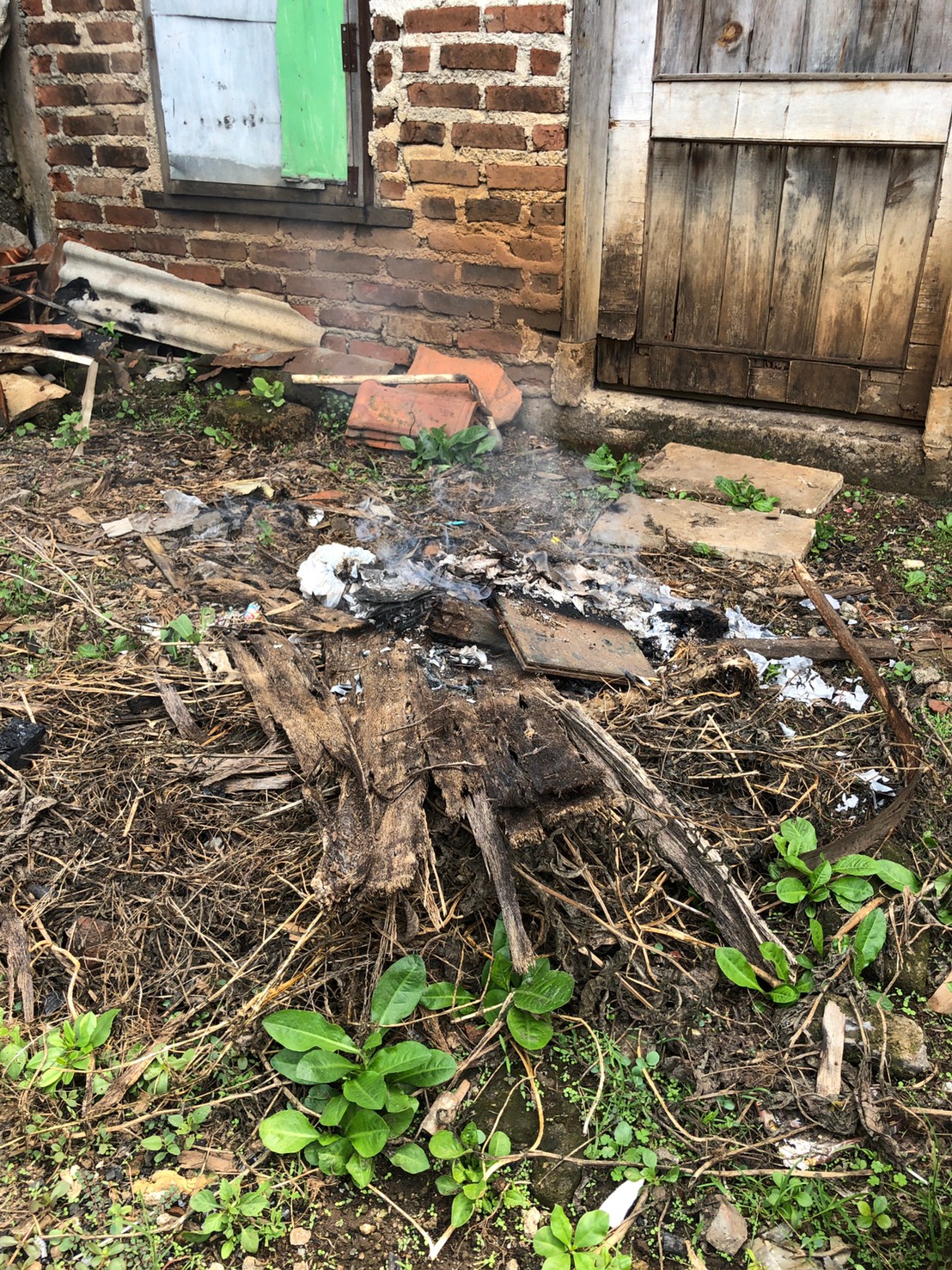 |
| 1.2 |  | Level of direct dumping on land | High | - In areas without services, there is evidence that residents may regularly dump to land - It believed to be an important means of disposal to the surrounded area | 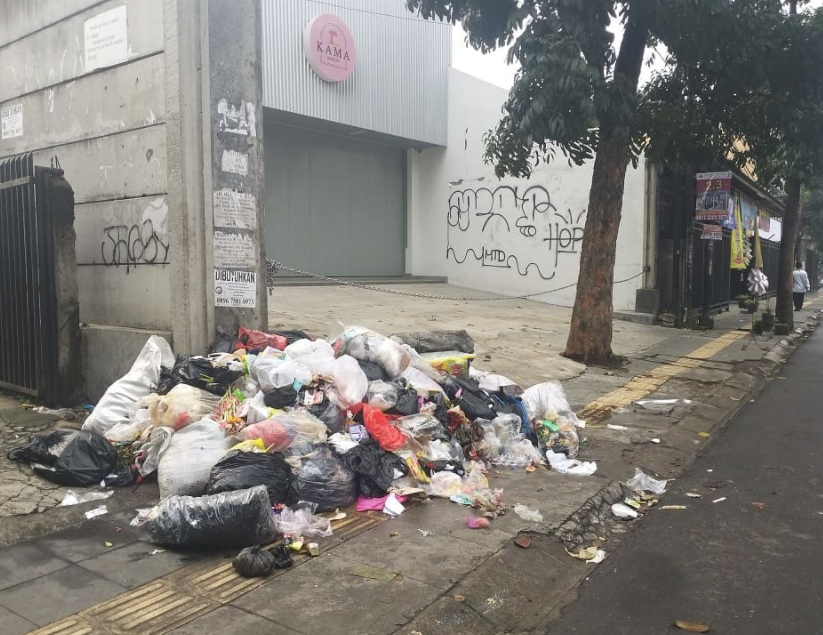 |
| 1.3 |  | Level of direct dumping in drains | Medium | - There is evidence of that residents may dump  their waste but not the majority | 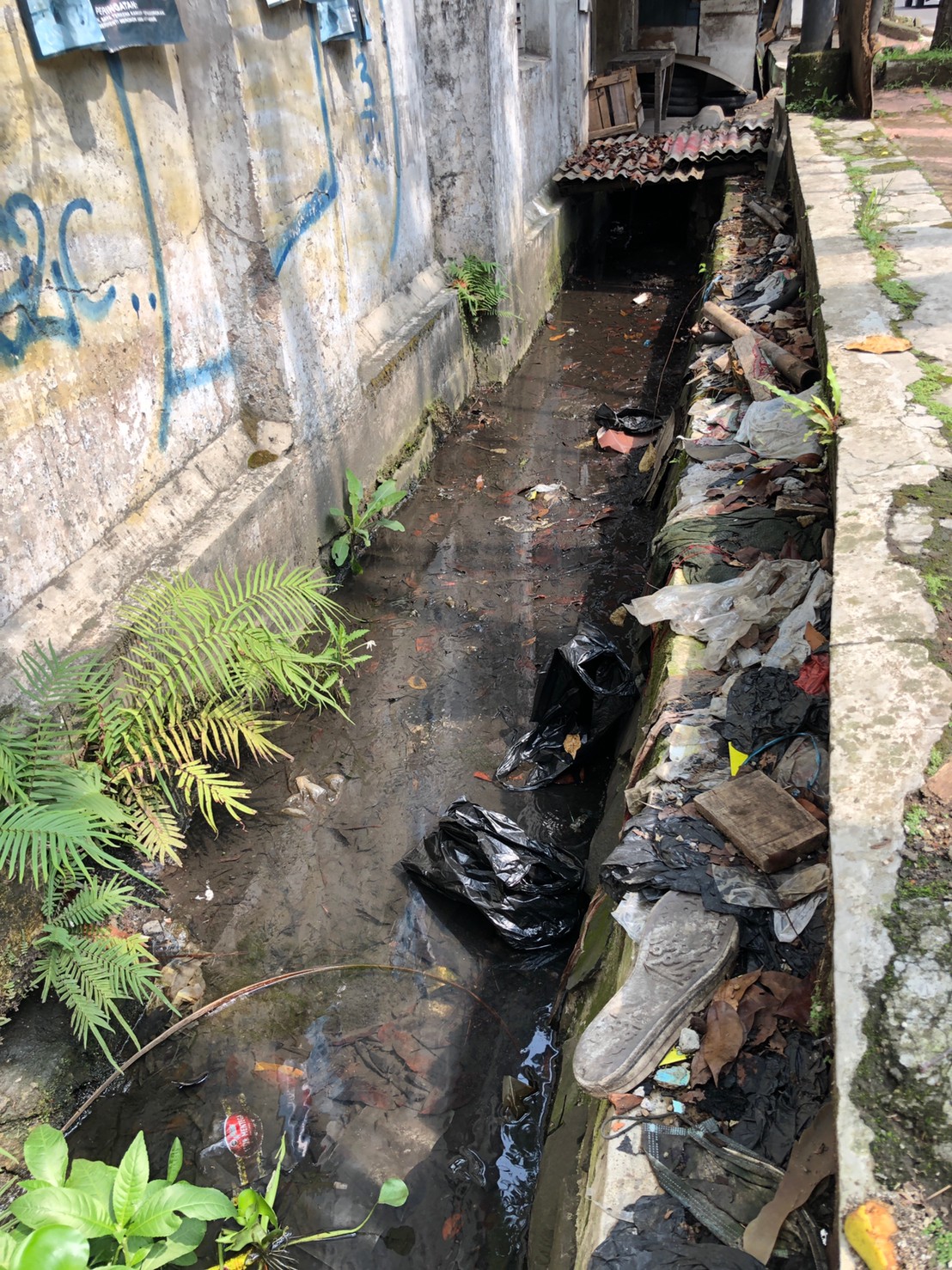 |
| 1.4 |  | Level of direct dumping in water system | High | - In areas without services, majority disposed  of the waste, which they have access to water body | 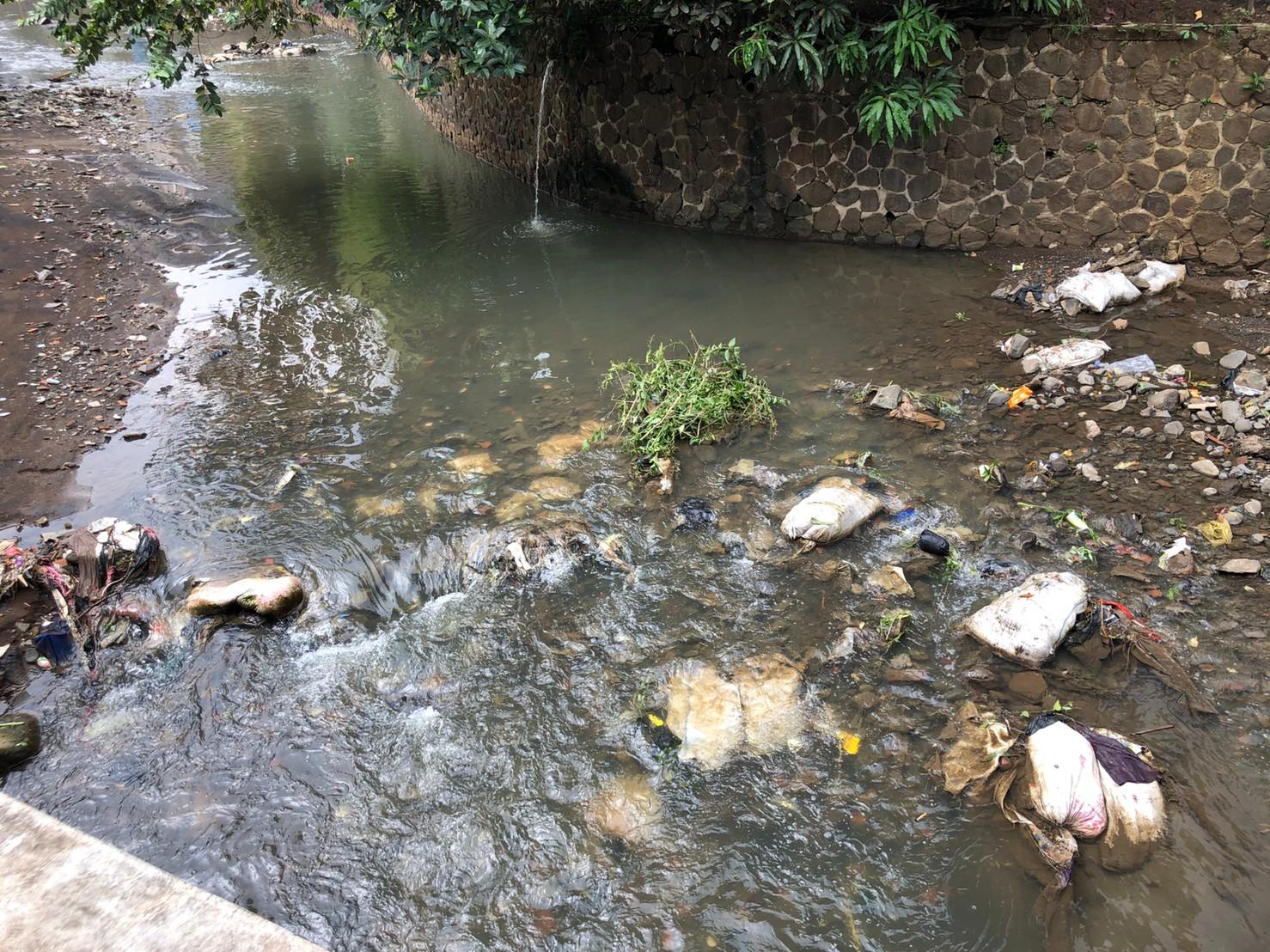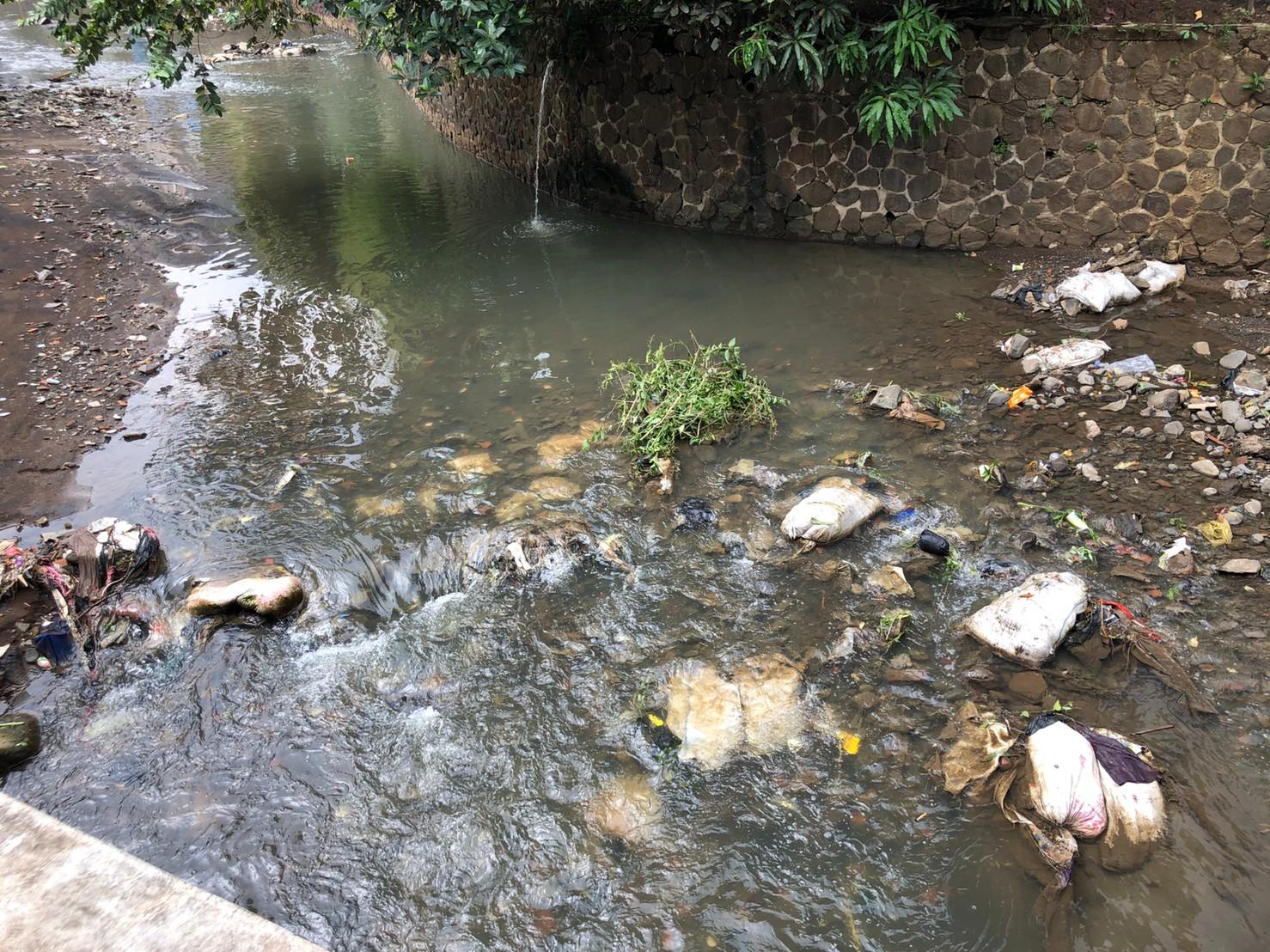 |
| 2.1 | Fate of plastic waste leaked during collection and transportation | Level of plastic to land | Low | - Throughout all the study area, there is evidence  of small quantities of plastic remaining on land | 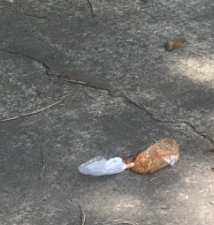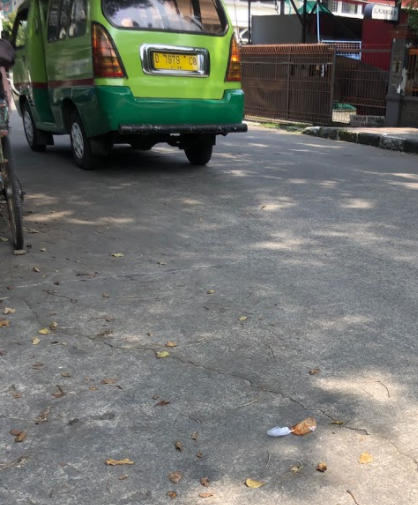 |
| 2.2 |  | Level of plastic to drains | Medium | - Majority of area, there is evidence  of small quantities of plastic leakage - Although a minority of areas show evidence  of large quantities of leakage | 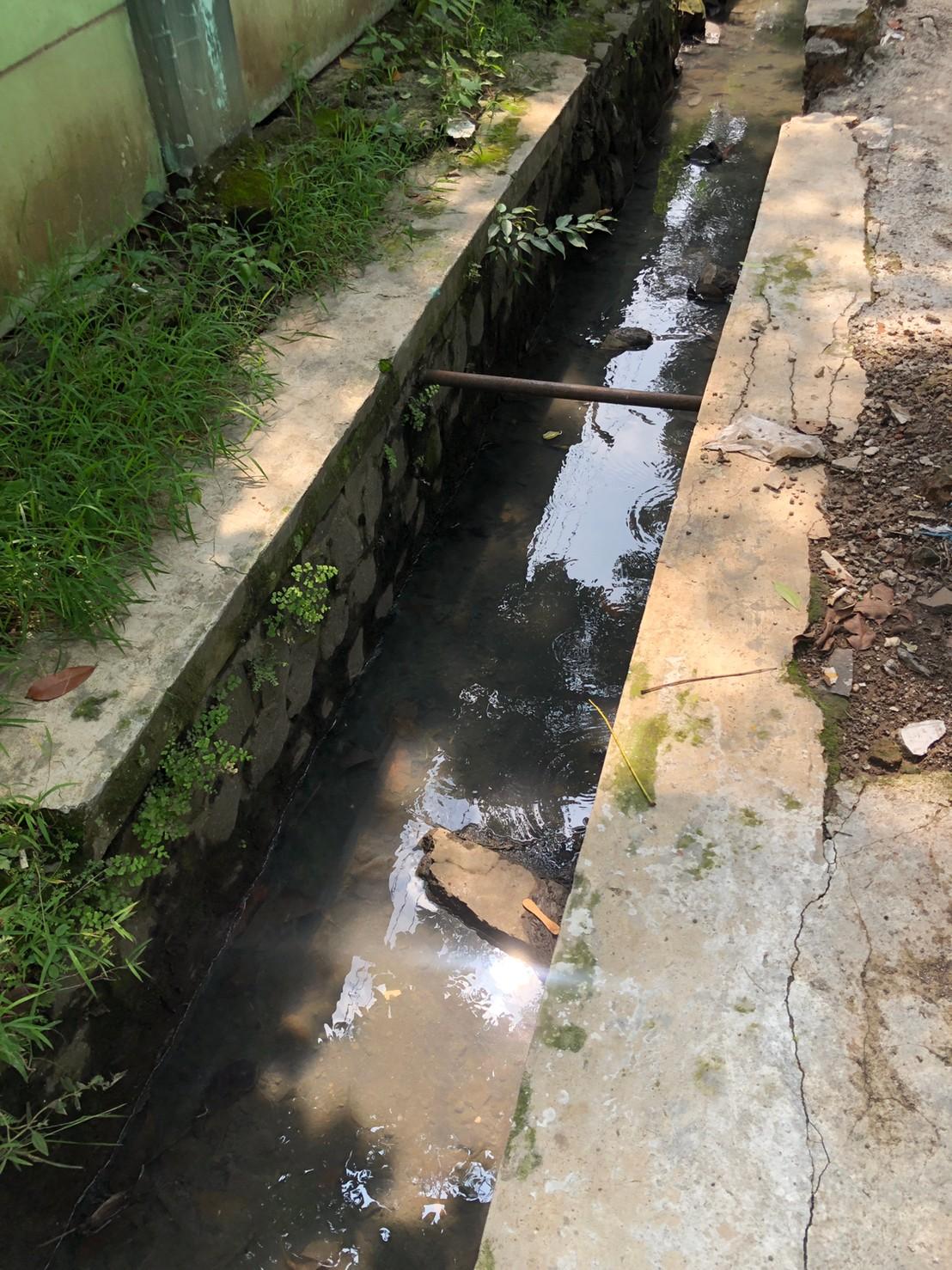 |
| 2.3 |  | Level of plastic to water systems | High | - Majority of the area close proximity (<1 km)  to the water system - Vegetation on the banks of the water systems is sparse throughout large parts of the study area. | 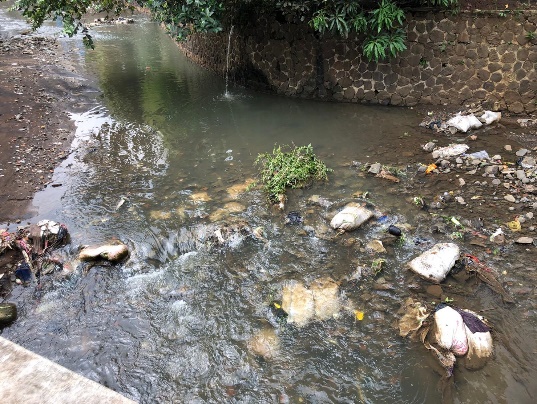 |
| 3.1 | Fate of plastic waste leaked from formal sorting | Level of plastic openly burnt | None | - No evidence of open burning | Assessed during field observation |
| 3.2 |  | Level of direct dumping on land | Very Low | - It is believed to the rare occurrence and unintentionally enter the surrounding land | 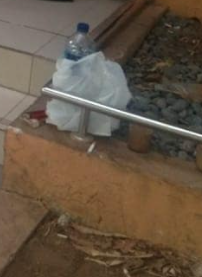 |
| 3.3 |  | Level of direct dumping in drains | Very Low | - It is believed to the rare occurrence and unintentionally enter the drains | Assessed during field observation |
| 3.4 |  | Level of direct dumping to water systems | Very Low | - Very few sorting facilities are <500 m to water systems and it is believed controlled | 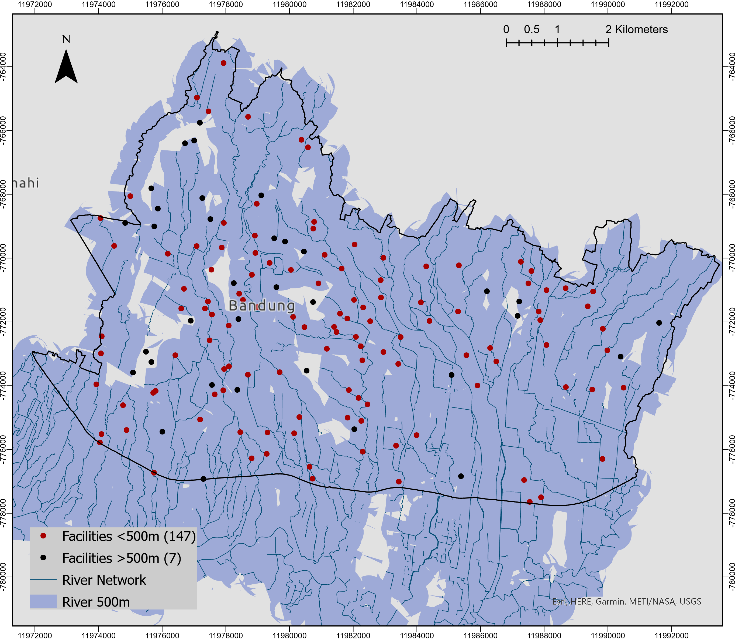 |
| 4.1 | Fate of plastic waste leaked from informal sorting | Level of plastic openly burnt | Low | - There is sporadic evidence, but it is believed to be a rare occurrence | 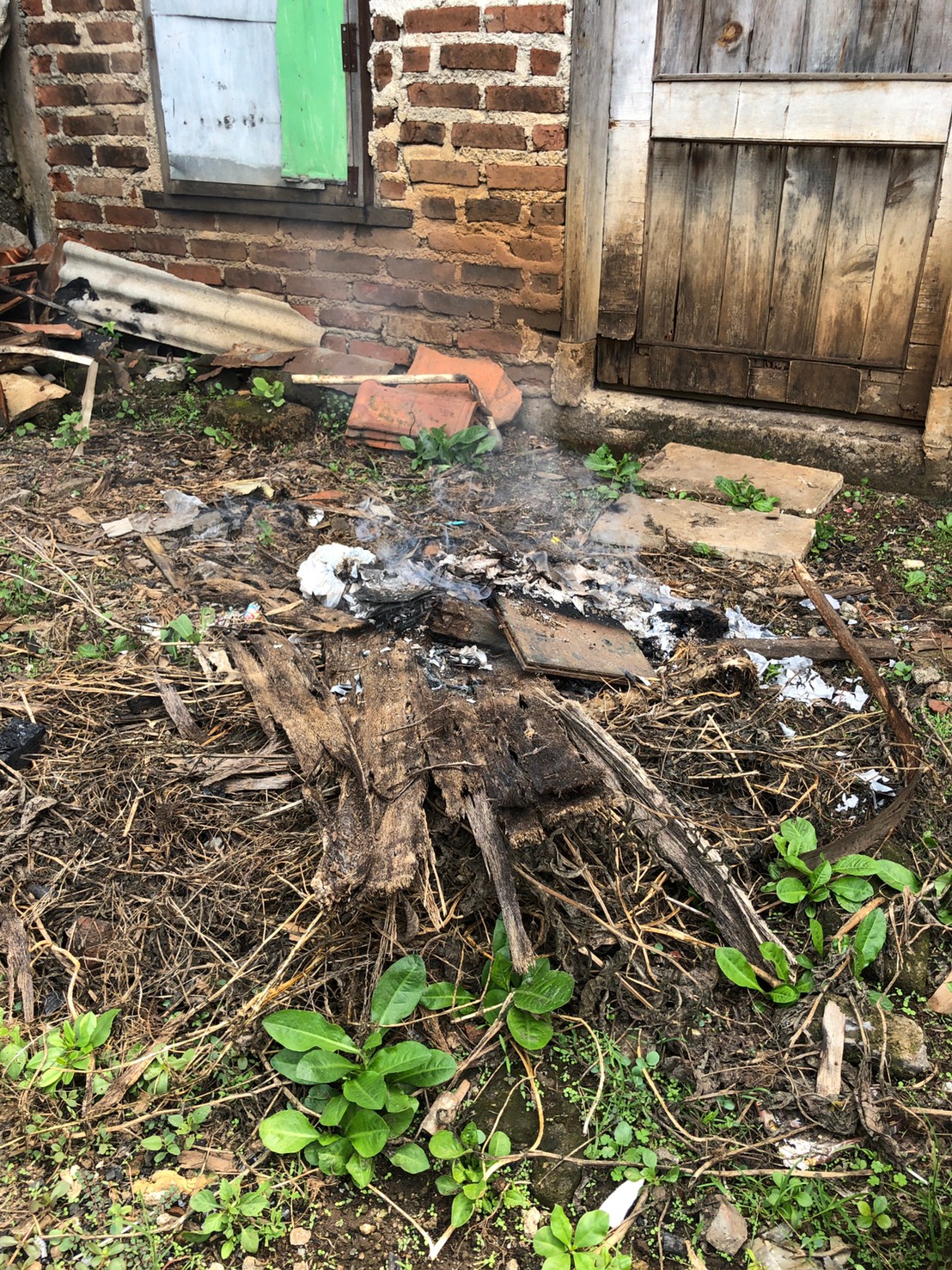 |
| 4.2 |  | Level of direct dumping on land | Medium | - There is sporadic evidence that a minority of sorting facilities may regularly dump their rejects to the surrounding areas, and street sweeping occasionally occurs in the vicinity | 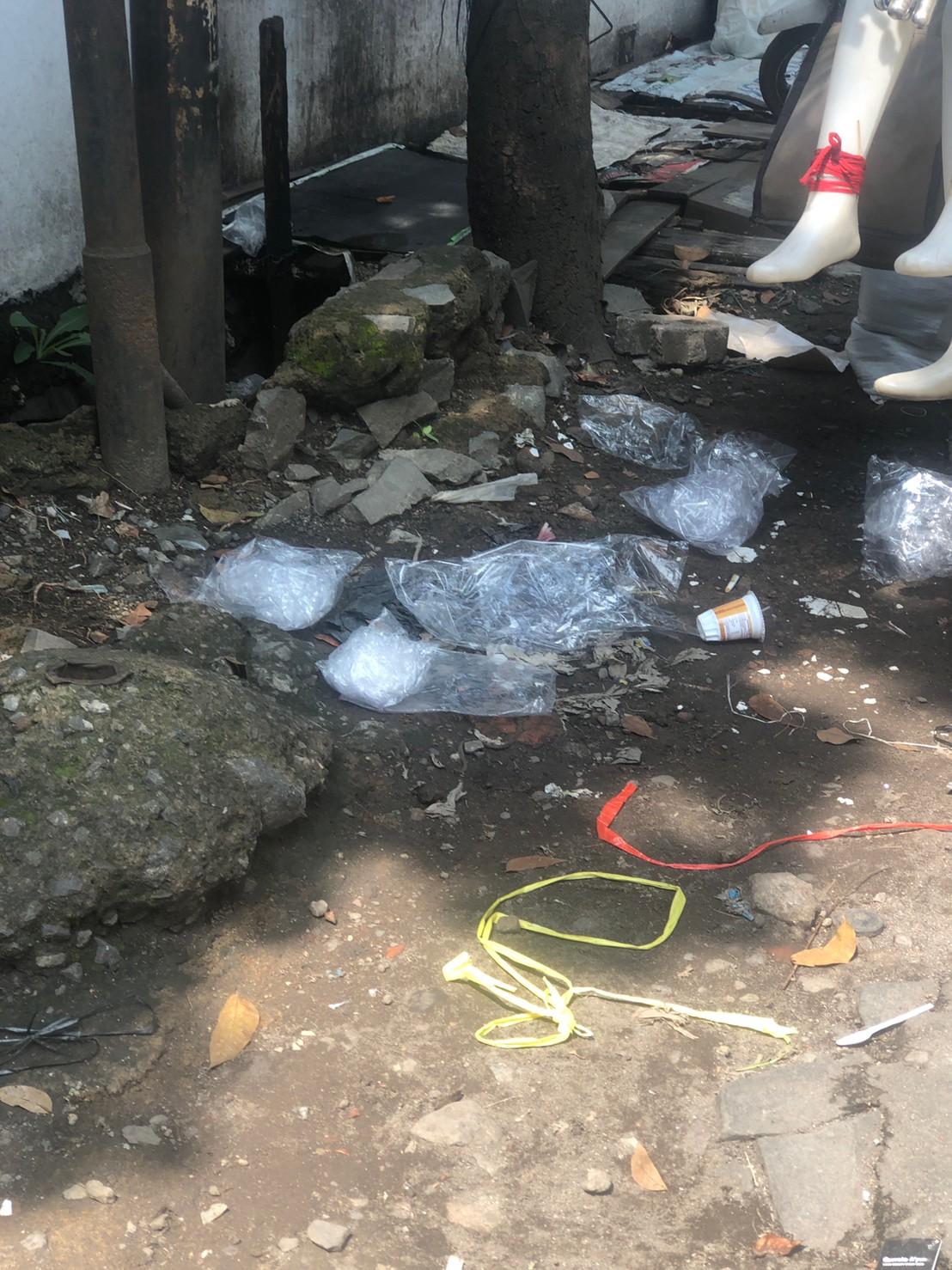 |
| 4.3 |  | Level of direct dumping in drains | Medium | - Minority of sorting facilities may regularly dispose their rejects to drains, and no active cleaning | 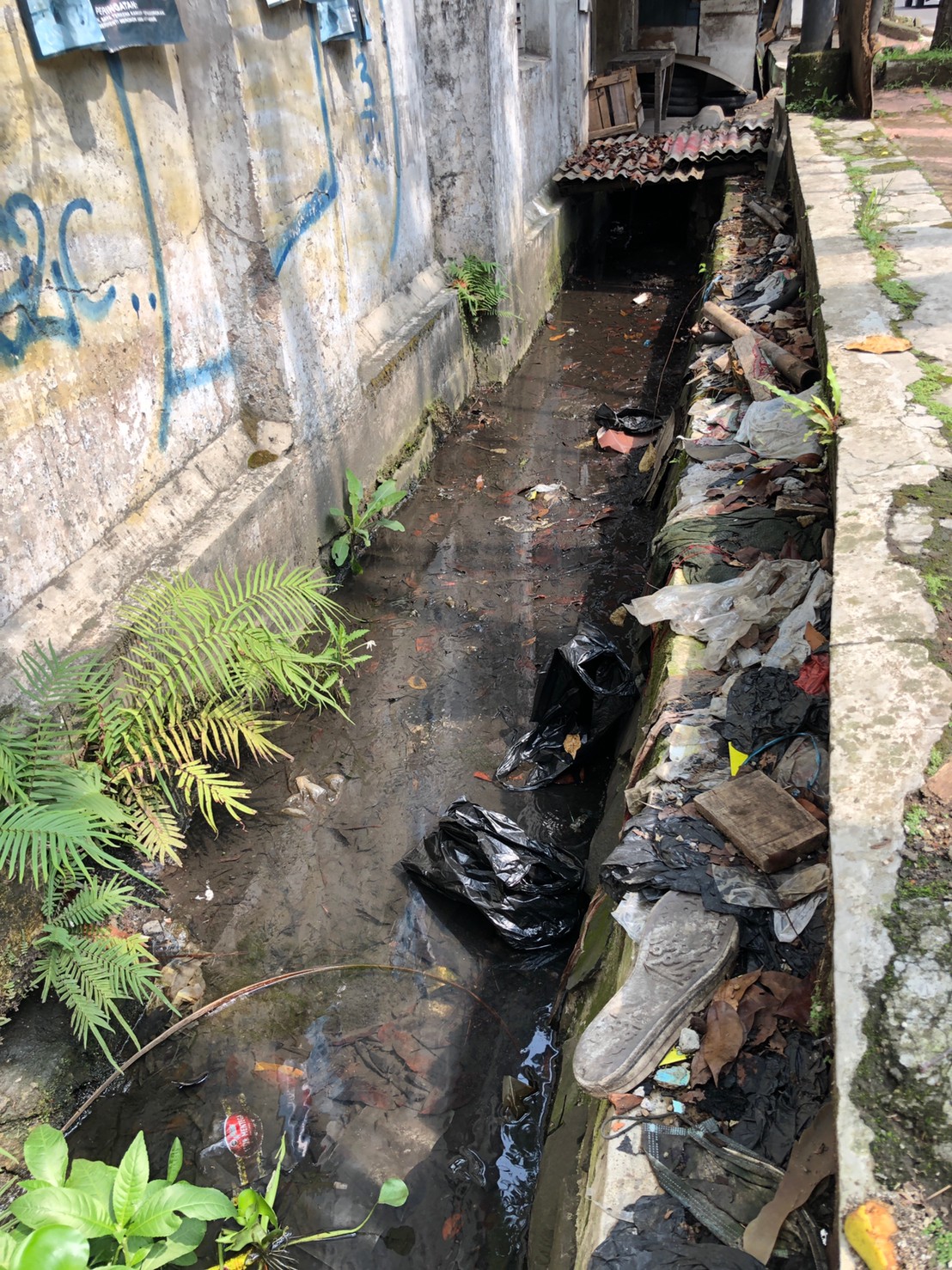 |
| 4.4 |  | Level of direct dumping to water systems | Low | - Very few sorting facilities are <500 m to water systems | Assessed during field observation |
| 5.1 | Fate of plastic waste leaked from disposal facilities | Level of plastic to land | High | - There is evidence of plastic waste remaining on land,  - In fact, the landfill has reached its capacity | Assessed during field observation |
| 5.2 |  | Level of plastic to drains | High | - There is evidence of plastic waste entering storm drains in the vicinity of the landfill | 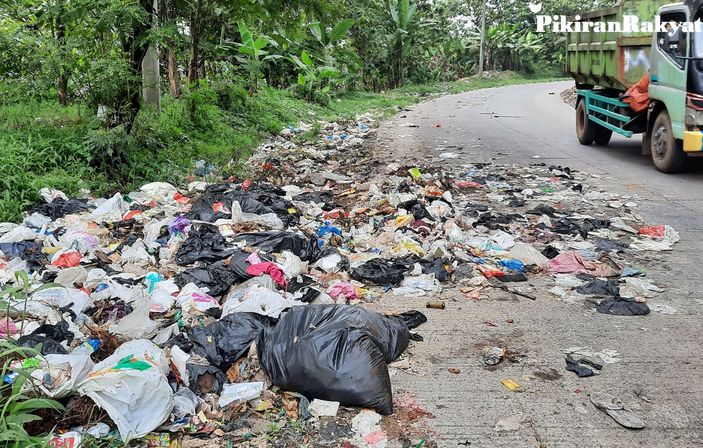[10] |
| 5.3 |  | Level of plastic to water systems | High | - It is close to the water system (<1 km) - There is the vegetation on the banks  of the water system - Often leak during rain due to landslide and flood | 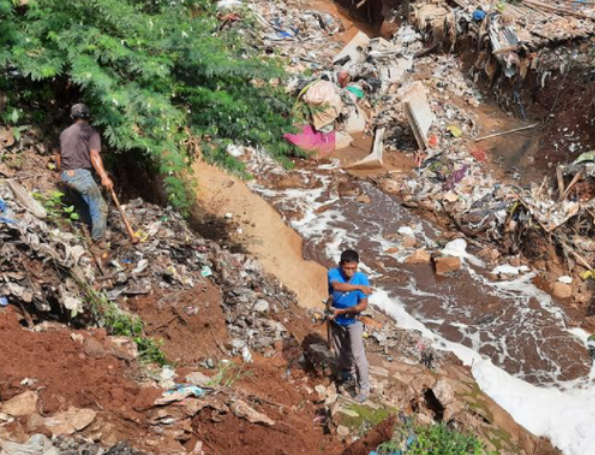[11] |

Table S6. Sample size for Interviews and Field Observations in Jakarta and Bandung

| **Data Collection Methods** | **Sample** | **Sample Size** | | **Descriptions** |
| --- | --- | --- | --- | --- |
|  |  | ***Jakarta*** | ***Bandung*** |  |
| **Interviews** | Waste municipality | 1 | 1 | Governmental agency that works for the respective cities. |
|  | Households | 44 | 97 | In Bandung, 95% confidence level and 10% of error. |
|  | Non-households | 3 | 14 | Commercial areas. |
|  | Waste recycler (formal) | 1 | 1 | Governmental agency that works for the respective cities. |
|  | Informal waste recycler | 1 | 1 | Private sector who responsible for the most recycling activity in the respective cities. |
| **Field Observations** | **1. Leakage** |  |  |  |
|  | 1.1 Collection services | 44 | 30 | One observation of each sub-district. |
|  | 1.2 Informal value chain collection | 44 | 30 | One observation of each sub-district. |
|  | 1.3 Formal sorting | 3 | 1 | Waste banks. |
|  | 1.4 Informal sorting | 3 | 3 | Informal sorting facility. |
|  | 1.5 Transportation to disposal | 44 | 30 | One observation of each sub-district. |
|  | 1.6 Disposal facilities | 2 | 7 | In Jakarta, 2 waste transfer stations. In Bandung, 6 waste transfer stations and a landfill. |
|  | 1.7 Drains | 44 | 30 | One observation of each sub-district. |
|  | **2. Fate** |  |  |  |
|  | 2.1 Uncollected plastic waste | 44 | 30 | One observation of each sub-district. |
|  | 2.2 Collection and transportation | 44 | 30 | One observation of each sub-district. |
|  | 2.3 Formal sorting | 3 | 1 | Waste banks. |
|  | 2.4 Informal sorting | 3 | 3 | Informal sorting facility. |
|  | 2.5 Disposal facilities | 2 | 7 | In Jakarta, 2 waste transfer stations. In Bandung, 6 waste transfer stations and a landfill. |

Reference

1. Adnan, M. S. G., Dewan, A., Zannat, K. E., & Abdullah, A. Y. M. The use of watershed geomorphic data in flash flood susceptibility zoning: a case study of the Karnaphuli and Sangu River basins of Bangladesh. Nat. Hazards 99, 425-448 (2019). <https://doi.org/10.1007/s11069-019-03749-3>

Putri, A.R., Fujimori, T. & Takaoka, M. Plastic waste management in Jakarta, Indonesia: evaluation of material flow and recycling scheme. *J Mater Cycles Waste Manag* **20,**2140–2149 (2018) <https://doi.org/10.1007/s10163-018-0753-2>

Sinulingga, B. Gunungan Sampah di TPST Bantargebang Capai Ketinggian Maksimal, Longsor Hantui Warga dan Pemulung (2021, Sep 17). [Web article] <https://www.liputan6.com/news/read/4660355/gunungan-sampah-di-tpst-bantargebang-capai-ketinggian-maksimal-longsor-hantui-warga-dan-pemulung>

UPTD DLH Provinsi DKI Jakarta. Tempat Pengelolaan Sampah Terpadu Bantargebang (2021). [web portal] <https://upstdlh.id/tpst/index>

Sukwika, T. and Noviana, L. Status Keberlanjutan Pengelolaan Sampah Terpadu di TPST‐Bantargebang Bekasi: Menggunakan
Rapfish dengan R Statistik. *Jurnal Ilmu Lingkungan*, **18**(1), 107‐118 (2020) <https://doi.org/10.14710/jil.18.1.107-118>

Weather Base. Jakarta, Indonesia (2021). [Web portal] <https://www.weatherbase.com/weather/weather-summary.php3?s=74769&cityname=Jakarta,+Indonesia>

Puspita, R. DPRD Jawa Barat: TPA Sarimukti Kelebihan Kapasitas (2021, June 21). [Web article] <https://repjabar.republika.co.id/berita/qv125t428/dprd-jawa-barat-tpa-sarimukti-kelebihan-kapasitas>

Romli, M. Jembatan Ambruk, Puluhan Warga Kampung Cidanas Bandung Barat Terisolir (2021, June 6). [Web article] <https://priangantimurnews.pikiran-rakyat.com/jawa-barat/pr-1222013937/jembatan-ambruk-puluhan-warga-kampung-cidanas-bandung-barat-terisolir?page=2>

Weather Base. Bandung, Indonesia (2021). [Web portal] <https://www.weatherbase.com/weather/weather-summary.php3?s=18769&cityname=Bandung,+Indonesia>

Arifianto, B. Tata Kelola TPA Sarimukti Bandung Barat Buruk, Tumpukan Sampah di Jalan Masih Ada padahal Pengangkutan Normal (2021, November 8). [Web article] <https://www.pikiran-rakyat.com/bandung-raya/pr-012972204/tata-kelola-tpa-sarimukti-bandung-barat-buruk-tumpukan-sampah-di-jalan-masih-ada-padahal-pengangkutan-normal?page=3>

Nurulliah, N. TPA Sarimukti Kritis dengan Tujuh Titik Longsor, DLH Jawa Barat Mohon Perhatian (2021, June 4). [Web article] <https://www.pikiran-rakyat.com/bandung-raya/pr-012001772/tpa-sarimukti-kritis-dengan-tujuh-titik-longsor-dlh-jawa-barat-mohon-perhatian>
